# Tracing the influence of Mediterranean climate on Southeastern Europe during the past 350,000 years

**DOI:** 10.1038/srep36334

**Published:** 2016-11-08

**Authors:** Igor Obreht, Christian Zeeden, Ulrich Hambach, Daniel Veres, Slobodan B. Marković, Janina Bösken, Zorica Svirčev, Nikola Bačević, Milivoj B. Gavrilov, Frank Lehmkuhl

**Affiliations:** 1 Department of Geography, RWTH Aachen University, Templergraben 55, 52056, Aachen, Germany; 2 BayCEER & Chair of Geomorphology, University of Bayreuth, 94450 Bayreuth, Germany; 3 Laboratory for Paleoenvironmental Reconstruction, Faculty of Sciences, University of Novi Sad, Trg Dositeja Obradovića 2, 21000 Novi Sad, Serbia; 4 Institute of Speleology, Romanian Academy, Clinicilor 5, 400006 Cluj-Napoca, Romania; 5 Interdisciplinary Research Institute on Bio-Nano-Science of Babes-Bolyai University, Treboniu Laurean 42, 400271 Cluj-Napoca, Romania; 6 Department of Geography, Faculty of Natural Sciences and Mathematics, University of Kosovska Mitrovica, Lole Ribara 29, 38220, Kosovska Mitrovica, Serbia

## Abstract

Loess-palaeosol sequences are valuable archives of past environmental changes. Although regional palaeoclimatic trends and conditions in Southeastern Europe have been inferred from loess sequences, large scale forcing mechanisms responsible for their formation have yet to be determined. Southeastern Europe is a climatically sensitive region, existing under the strong influence of both Mediterranean and continental climates. Establishment of the spatial and temporal evolution and interaction of these climatic areas is essential to understand the mechanisms of loess formation. Here we present high-resolution grain-size, environmental magnetic, spectrophotometric and geochemical data from the Stalać section in the Central Balkans (Serbia) for the past ~350,000 years. The goal of this study is to determine the influence of the Mediterranean climate during this period. Data show that the Central Balkans were under different atmospheric circulation regimes, especially during Marine Isotope Stages 9 and 7, while continental climate prevailed further north. We observe a general weakening of the Mediterranean climate influence with time. Our data suggest that Marine Isotope Stage 5 was the first interglacial in the Central Balkans that had continental climate characteristics. This prominent shift in climatic conditions resulted in unexpectedly warm and humid conditions during the last glacial.

Knowledge of past climate variability based on the study of palaeoclimate archives may help clarify past climatic forcing mechanisms and help predict the extent of future climate change^1^,^2^. In Southeastern Europe, loess-palaeosol sequences are one of the most important and often the only available terrestrial archives of Quaternary palaeoclimate dynamics^3^,^4^. Loess-palaeosol sequences from the Middle and Lower Danube Basins (Fig. 1) have been the focus of recent research on the Quaternary in Europe^5^,^6^,^7^,^8^,^9^,^10^,^11^,^12^, and as a result, the understanding of the Late Quaternary climate and environmental conditions in the region have been fundamentally improved. Although the studies of loess sequences in Southeastern Europe have provided local and regional information of past environments, they have not focused on identifying large scale forcing mechanisms and the climatic conditions responsible for loess-palaeosol sequences formation, particularly whether or not a Mediterranean climatic influence may have existed in the region. One of the main reasons for this lack of data is that the Middle and Lower Danube Basins are influenced by three different climatic systems: Atlantic, continental and Mediterranean. It is challenging to distinguish which of these climate systems may have dominated during the recent past. Also, the loess-palaeosol sequences as palaeoenvironmental records in the Middle and Lower Danube Basins are generally limited in terms of their climate sensitivity due to the overall effect of prolonged dryness in the region^3^,^5^,^8^,^13^, which is an additional limiting factor in establishing the relative influence of associated atmospheric systems.

The Central Balkans (Fig. 1) are situated in a transition area between the temperate-continental climate in the north and the Mediterranean climate in the south. The area is currently more influenced by the Mediterranean climate than are the Middle and Lower Danube Basins^14^. Although palaeoclimate records from the Central Balkans have been poorly investigated^14^,^15^,^16^, this area may be key to understanding past changes between these two climate zones. To address this, we conducted a high-resolution multi-proxy investigation of the Stalać section in the Central Balkans (Serbia; Fig. 1). For this site, we examined grain-size, environmental magnetism, and other spectrophotometric and geochemical proxies to reconstruct past climatic and environmental dynamics for the past 350,000 years. Grain-size composition is one of the most frequently used palaeoenvironmental proxies of loess sequences to infer changes in aeolian dynamics, sources of loess and pedogenesis^17^,^18^,^19^,^20^. Based on the principle of post-depositional formation of ultrafine magnetic particles during pedogenesis, environmental magnetism has also been successfully applied as a palaeoclimatic proxy in Eurasian loess^6^,^9^,^21^. Color variations in loess research have also been used as a proxy for variations in mineral concentrations and as an indicator of past pedogenesis^6^,^22^. Finally, the composition of geochemical elements has been successfully applied to establish temporal weathering intensity in Southeastern Europe^5^,^23^ and also used to establish the provenance of loess in same region^12^,^24^.

The main goal of this study is to better understand the degree to which Mediterranean and continental climate zones have influenced this part of Southeastern Europe, by comparing loess deposits in the Central Balkans and palaeo-archives from the surrounding areas (the continental Middle Danube Basin, the South Balkans and the Mediterranean itself). Differences observed in the characteristics of loess sediments in these regions may help identify past spatial variations in the dominant atmospheric systems over Southeastern Europe. The interpretation of the relationships between the Mediterranean and continental climate zones would be a significant step toward a wider understanding of the past atmospheric circulation patterns over continental Europe at large. Past research has suggested that Southeastern Europe has been progressively influenced by drier continental climate over time^25^. Thus, past climatic intervals, proposed as the best analogues to modern conditions according to astronomically driven orbital changes on global scale (e.g. Marine Isotope Stages (MIS) 11 and 19^1^) may not strictly be representative in this region, since those periods experienced more humid conditions. In such a region with no comparable analogues, research on the interaction between large scale climate systems and their feedback mechanisms is important to the understanding of future climate change. Besides addressing regional teleconnections, we also evaluate the influence of global climate changes on the continental part of Southeastern Europe.

## Results

### Stratigraphy and chronology

Figure 2 presents the Stalać composite stratigraphic profile built from five sampled sub-sections; for details see the Supplementary material. The labeling of the stratigraphic units follows the established scheme for the Danube loess stratigraphy^3^. The age model is based on the correlation of odd-numbered MIS to phases of soil formation, and it is confirmed by tephra chemistry and luminescence dating (details are presented in section 5 in Supplementary material).

The composite profile (Fig. 2) shows the sedimentological characteristics of loess-palaeosol sequences accumulated during the past ~350,000 years. The stratigraphic profile commences with a L4 loess unit (uncovered only 0.4 m). In the top of this unit is a strongly developed, brown-red Cambisol with vertic characteristics and a thickness of ca. 1.25 m (S3). This palaeosol is overlain by a layer of typical loess, ranging in thickness from 1.65 to 3 m (L3). From 3.0 to 3.45 m, another Cambisol with vertic characteristics is exposed (S2), but this one is less strongly developed than the S3 palaeosol. The S2 palaeosol is followed by a 5.4 m thick, pale yellow loess layer (L2). A volcanic tephra layer is intercalated within the L2 loess (4.85–4.9 m). The L2 loess ends in a 0.85 m thick Kastanozem-type palaeosol (S1), overlain by a brown-yellow loess layer, approximately 1.30 m thick (L1LL2). This layer terminates in a 1.1 m thick palaeosol (L1SS1SSS2), covered by a 0.7 m thick but darker loess layer (L1SS1LLL1). Microscopic investigation of this layer shows high abundance of well-preserved glass shards, hinting at the presence of a cryptotephra horizon within this layer. Above, another palaeosol horizon (12.85–13.65 m) is developed (L1SS1SSS1). A brownish loess layer is exposed from 13.65–14.7 m (L1LL1), on top of which is developed the modern topsoil (S0). The upper part of the modern soil has been heavily influenced by modern agriculture, therefore only the lowermost 30 cm have been sampled. Material in Profile 1 was deposited on the different geomorphological conditions than in profiles 2–5, and therefore it is challenging to compare the sedimentation rates from profile 1 to the rest; further details are available in Supplementary material.

### Particle size properties and environmental magnetism

The entire section is comprised of loess and is silt-dominated, particularly coarse silt (20–63 μm sized particles). When compared to other Eurasian loess-palaeosol sequences the Stalać section has a high sand (>63 μm sized particles) content (Supplementary Fig. 5). The grain-size distribution (see Supplementary Fig. S9) suggests that only the L1SS1LLL1 layer is not unaltered loess; instead, it may have been affected by special sedimentological conditions, showing a unique peak in sand contribution. Generally, all palaeosols are comparatively enriched in clay (<2 μm particles) with highest values in S3 (maximum of 28%); the various intervening loess units contain less clay (minimum of 4.7%; Fig. 2). The U ratio (coarse/fine silt (16–44/5.5–16 μm); Fig. 2) has been used as a proxy for wind strength^26^. The mass specific magnetic susceptibility (χ; Fig. 2) varies between 82.1*10^−8^ and 371.8*10^−8 ^m^3^/kg (average 189*10^−8 ^m^3^/kg) almost an order of magnitude larger than for other loess deposits in the Danube Basin, Central Asia and China^6^. Maximum χ values occur in the L2 tephra layer, whereas high values occur in the younger parts of L3 and L1. Minimum χ values occur in the basal L4 unit. The L2 (excluding the tephra layer) also has low values of χ. The frequency dependent magnetic susceptibility (χ_fd_) varies between 3.1 to 7.3% (average 4.6%). This proxy follows the lithostratigraphy (Supplementary Fig. 6), having higher values in palaeosols (maximum in S3) and lower values in the intervening loess units (minimum in L2) but not reaching the average level (higher of 10%) of interglacial palaeosols in common loess-palaeosol sequences^6^.

### Bulk sediment geochemistry

The sediment is dominated by SiO_2_ although with a large range, oscillating between 39.87 and 66.4% (average 59.57%); these values are typical for loess deposits^12^,^24^. Also Al_2_O_3_ (average 15.95%), CaO (in a wide range from 0.81–36.36%; average 9.22%), FeO (average 6.34%) and MgO (average 3.76%) are strongly represented in the sediments, and this, too, is similar to other European loess-palaeosol sequences^12^,^24^. All other elements comprise less than 3%. A unique characteristic of the Stalać loess is the high concentration of Ni and Cr. Ni values range from 46.5 to 172.6 ppm (average 110.3 ppm) and Cr ranges from 99.4 to 310.6 ppm (average 210.6 ppm). Generally, Ni and Cr concentrations exhibit wide variations between and within different stratigraphic units. High values are recorded in L1 and L3, whereas other units have lower concentrations (Fig. 2). Supplementary Table 1 presents Ni and Cr values for alluvium from the nearby rivers. The Zapadna Morava alluvium has the highest concentrations, while the Južna Morava alluvium contains lower amounts of Cr and Ni.

### Tephrochronology

Based on visual field observations, as well as trends in sedimentological data, two main tephra layers have been identified for the studied time-interval (Fig. 3 and Supplementary Fig. 7). Whereas glass shards from the visible tephra within L2 are completely altered and thus unsuitable for geochemical tracing of the volcanic source, the chemical investigation of glass shards from upper tephra in L1SS1LLL1 layer is presented in Supplementary Table 3. With average SiO_2_ content of 59.25 wt%, associated with 18.14 wt% Al_2_O_3_, 7.34 wt% K_2_O, 5.39 wt% Na_2_O, 2.94 wt% FeO and 1.84 wt% CaO, the major oxide data confirm that this perialkaline trachitic cryptotephra layer is yet another occurrence of the regionally widespread Campanian Ignimbrite/Y-5 tephra^27^,^28^,^29^,^30^ (Supplementary Fig. 9 and Supplementary Table 3).

### Spectrophotometric results

The spectrophotometric data from the Stalać composite profile is presented in Fig. 2 and Supplementary Fig. 6. Lightness (L*; see Methods) tends to indicate the production of biomass during the sediment formation. L* values (ranging from 52.66 to 75.13) are higher in loess and lower in palaeosol units. However, loess layers L1LL1, L1SS1LLL1 and L1LL2 are darker (lower L* values) than loess units L2 and L3. Redness (a*; red-green scale) correlates well with lithostratigraphy, having high values in palaeosols and low in loess units (except in L1SS1LLL1). Differences in colour between different palaeosols are clearly visible. The highest a* values occur in the S3 palaeosol (up to 6.82) and are somewhat lower in S2 (up to 5.48), while the values in S1, L1SS1SSS2 and L1SS1SSS1 do not exceed 5. The b* (blue-yellow) has its highest values in S3 (maximum 24.09) and S2 palaeosols, but lowest in S1, L1SS1SSS2 and L1SS1SSS1 palaeosols (minimum value 16.91). The modern soil has very low L* and high a* and b* values.

## Discussion

**Figure 1:**
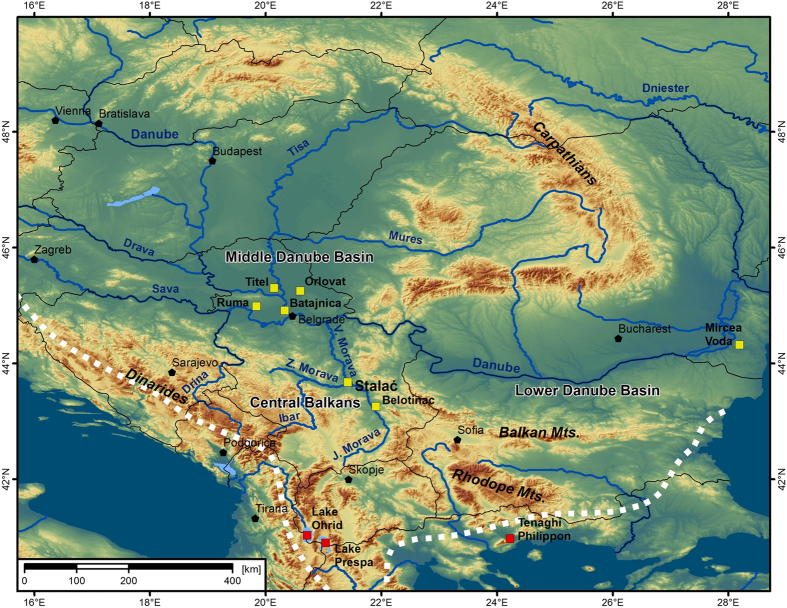
Map of the Balkan Peninsula, Middle and Lower Danube Basins, showing key loess-palaeosol sequences (yellow rectangles; Stalać (this study), Ruma^26^, Titel^36^, Batajnica^5^,^6^,^25^, Orlovat^10^,^37^, Belotinac^15^,^16^, Mircea Voda^5^,^6^,^25^) and lacustrine records (red rectangles; Ohrid^34^, Prespa^35^ and Tenaghi Philippon^31^) discussed in this paper. The white dashed line represents the current northern limit of the Mediterranean climate. The map was generated using ArcGIS 10.2.2 (http://www.esri.com/software/arcgis/arcgis-for-desktop/free-trial).

To understand the significance of the presented data, it is important to realize that besides several available lacustrine records from the southern Balkan Peninsula (Tenaghi Philippon^31^, Ioannina^32^, Kopais^33^, Ohrid^34^ and Prespa^35^), other high resolution records in the interior are still missing in the Balkan region (Fig. 1). Comparing the climate evolution from the interior of the Balkans with records from Mediterranean climate areas, such as the Tenaghi Philippon lacustrine record^31^, can provide useful insights into the Mediterranean climate circulation patterns over the Balkans. Moreover, understanding the relations between Stalać and Lake Ohrid^34^ is of special importance. Because of its altitude of 693 m a.s.l. and its considerable distance from the sea (Fig. 1), this lake is currently under a modified Mediterranean climatic influence and is more sensitive to continental climate than are other lacustrine records in the Balkans. Finally, understanding of the relations between Stalać and the well-studied loess-palaeosol sequences in the Middle Danube Basin improves the understanding of the interplay between the Mediterranean and continental climates in Southeastern Europe during the Quaternary.

The geochemical properties of the Stalać loess-palaeosol sequence are characterized by high concentrations of Ni and Cr (Fig. 3 and Supplementary Fig. 11), values that unequivocally indicate surrounding river valleys (Južna, Zapadna and Velika Morava Rivers) as the source areas (for more details see Supplementary material). This is also supported by the higher contribution of the coarser grain-size particles, when compared to middle Danube basin^26^,^36^,^37^. Such geochemical and textural characteristics suggest limited or completely suspended contribution of the particles from the Danube catchment area, which is the main source area for the terrestrial sediments in the Middle and Lower Danube Basins. Despite the relative vicinity of the Danube River (Fig. 1), the absence (or very limited presence) of particles from its catchment area at the Stalać section indicates that the Central Balkans were not strongly influenced by the atmospheric circulation patterns and winds from the north. A sharp climatic border/transition between the southern limit of Middle Danube Basin and the area north of the Stalać section can explain the observed pattern.

**Figure 2:**
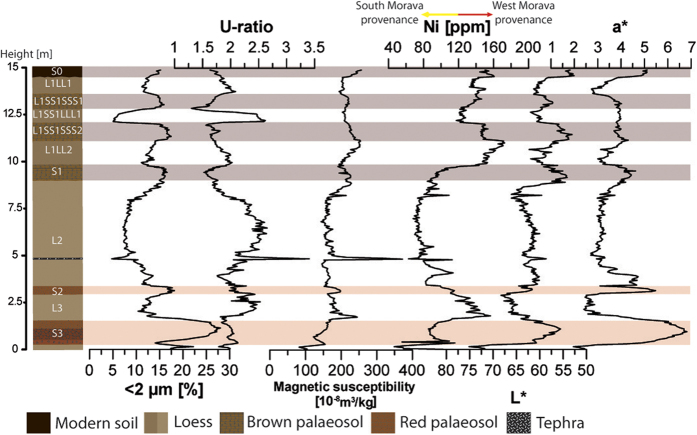
Clay fractions, U-ratio, χ, Ni contribution, L* and a* values (see Methods) related to pedostratigraphy of the composite profile from the Stalać section.

**Figure 3:**
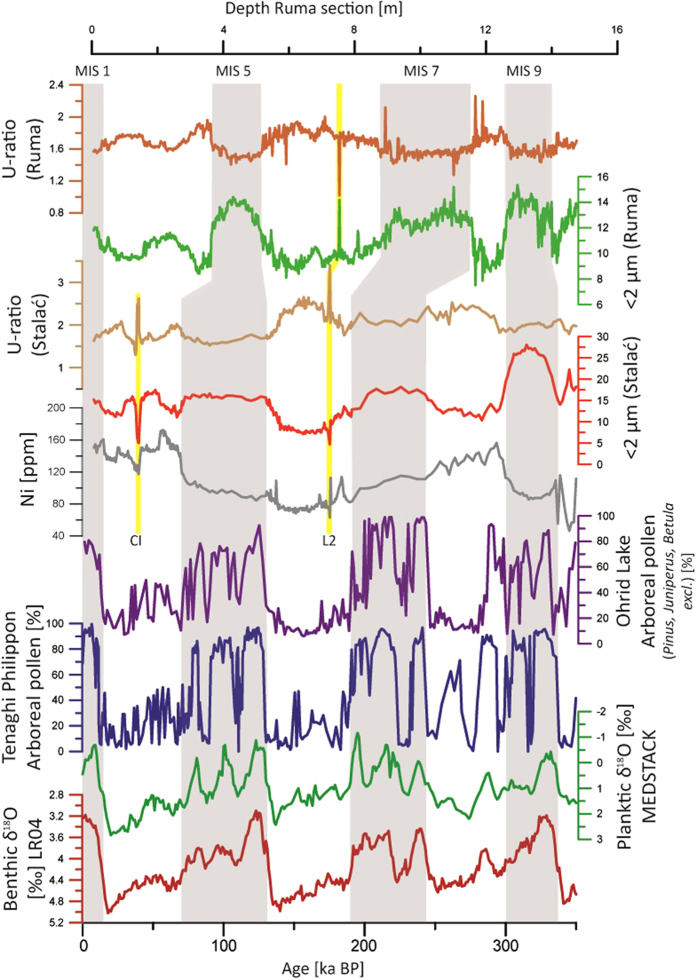
Direct comparison between the benthic δ^18^>O LR04 stack^59^, MEDSTACK planktic δ^18^O data^39^, arboreal pollen from Tenaghi Philippon^31^, arboreal pollen (*Pinus, Juniperus*and*Betula* are excluded) from the Lake Ohrid core^34^, U-ratio and <2 μm fractions from the Stalać section (plotted versus age, abscissa), U-ratio and <2 μm fractions from the Ruma section 26 (the only section with existing grain-size record spanning the last three glacial-interglacial cycle in Middle Danube Basin; plotted vs. depth). Note the differences between scales on the plots presenting Stalać and Ruma grain-size data. Tephra layers are marked with yellow lines; “CI” refers to the Campanian Ignimbrite/Y-5 tephra layer and “L2” refers to the L2 tephra layer.

Also, essential differences between these regions are observed in the palaeosol development within their respective sections, especially during MIS 9 and 7. For those interglacials, at the Stalać section the intercalated palaeosols are classified as Cambisols. These palaeosols are reddish in color (high a* values) and have high clay contents (Fig. 2). These data indicate that the Mediterranean climate influence was strong in this area during these interglacials (e.g. see references 5 and 6). Conversely, palaeosols in the Middle Danube Basin indicate the overall dominance of steppe or forest-steppe environments during the past ~350,000 years^38^, pointing to the influence of the continental climate during these interglacials^5^. Although MIS 7 and 9 in the Central Balkans were characterized by generally more precipitation than the Middle Danube Basin, summers were warmer and much dryer. Accordingly, during MIS 7 and 9, the Balkan Peninsula was under the strong influence of a sub-tropical anticyclone belt during the warm seasons, creating low precipitation during summer in the Mediterranean region, and thus, a Mediterranean-like climate dominated. Contrary, in the Middle Danube Basin these interglacials had relatively similar conditions to the present conditions, i.e., being under continental climate (cold winters and warm summers, with a precipitation maximum during the beginning of the warm period). Observed similarities in the Stalać data and the pollen data from Tenaghi Philippon^31^, Lake Ohrid^34^ and planktonic δ^18^O data from the Mediterranean (MEDSTACK)^39^ during MIS 9–MIS 6 (see Supplementary material) generally indicate similar climate conditions across the Balkans (Fig. 3).

**Figure 4:**
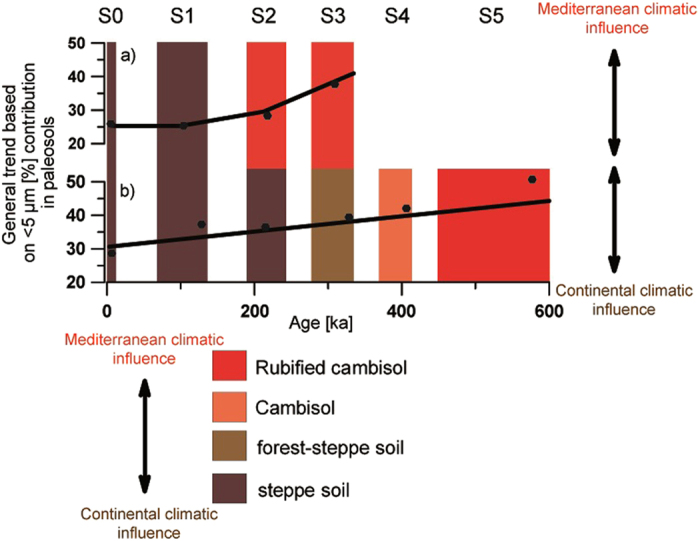
Progressive termination of Mediterranean influence over Southeastern Europe, as indicated by the succession of palaeosol types and <5 μm particles peak values in related palaeosols at (**a**) the Stalać section (Central Balkans) and (**b**) the Batajnica-Stari Slankamen spliced section^9^ (Middle Danube Basin).

However, although the Mediterranean-like climate was dominant during this time interval over the Central Balkans, differences in clay content and redness between palaeosol S3 (related to MIS 9) and S2 (related to MIS 7) clearly indicate the weakening of Mediterranean climate influence over time (Fig. 4); this trend is not observed in the other geoarchives of Mediterranean region (Fig. 3). Figure 4 presents a succession of palaeosol types from Cambisols towards steppe-like palaeosols, with a concomitant decrease in <5 μm particle peak values in the related palaeosols at the Stalać section (Central Balkans) and the Batajnica-Stari Slankamen (spliced) section^9^ (Middle Danube Basin). Although data from the Stalać section do not span the same time interval, a progressive diminution of Mediterranean climate influence can be observed within Southeastern Europe. This progressive evolution of continental climate (aridization and winter cooling) in the Middle and Lower Danube Basins since the Early Pleistocene was proposed to be caused by the uplift of the Alps, Carpathians and Dinarides^9^. For the Central Balkans, only the uplift of the Dinarides is likely to have played a major role in this climate shift. However, considering that the termination of the Mediterranean climate is more pronounced in the Central Balkans over the past 350,000 years, it is unlikely that the uplift of the Dinarides could have weakened the Mediterranean influence in the observed proportions. The present study demonstrates that the progressive evolution of the continental climate in the Middle Danube Basin and the progressive decrease of the Mediterranean climate influence in the Central Balkans are probably connected, although this trend is more expressed in the Central Balkans over the past 350,000 years. Additionally, a similar trend of increased continentality over time is observed in the Azov Sea region^40^. Thus the observed trend is not only a regional trend for the Middle and Lower Danube Basins, but may be a supra-regional climatic trend. A more complete understanding of mechanisms behind this pattern requires more long-term high-resolution data.

All the long palaeoclimate records for the Balkans^31^,^34^ (including Stalać) specify MIS 6 as the most intense glacial period with the most adverse conditions, and with a maximum of glacier expansion^41^,^42^,^43^. MIS 6 is also considered to represent the most intense glaciation in the rest of Europe^44^. Geochemical tracing of palaeo-river systems, based on the contributions of Ni and Cr (for more details see Supplementary material), shows that the L2 loess (equivalent to MIS 6) is the only such layer formed mainly by particles from the Južna Morava River valley (Fig. 2 and Supplementary Fig. 11). This suggests that dry conditions over the Zapadna Morava River catchment strongly reduced its discharge, whereas the discharge from the Južna Morava catchment was not so dramatically limited by the general dryness of this period (see Supplementary material). This is a clear indication of dry conditions over the entire interior of the Balkans, including high mountainous areas, suggesting that precipitation could not reach the interiors of the Balkans. Possible reasons for this may be (1) specially cold conditions that would have increase aridity, (2) the maximal expansion of glaciers in the Balkan mountain ranges^41^,^42^,^43^ causing a high-pressure barrier for the moist air from the south, and (3) increasing distance from the sea, the major source of humidity, due to the low sea level^45^ in this time interval.

Our study suggests a shift in the climatic and environmental conditions over the Central Balkans since MIS 5. The palaeosol S1 (equivalent to MIS 5) shows remarkably different features as compared to the older palaeosols at the Stalać section, indicating a limited Mediterranean climate influence. Similarities in the genesis of the S1 (MIS 5), L1SS1SSS2 and L1SS1SSS1 (MIS 3) pedocomplexes (Fig. 2) indicate similar conditions during the last interglacial and MIS 3 interstadials at the Stalać section. A high abundance of fine particles and low L* values from L1LL2 and L1LL1 loess layers (Fig. 2 and Supplementary Fig. 12) indicate that the transition from the last interglacial to the early last glacial was not sharp, and relatively mild conditions prevailed during the early and late last glaciation (Fig. 2). Most of the last glacial cycle at the Stalać section was relatively humid and mild compared to previous glacials. An exception is the L1SS1LLL1 layer, where volcanic glass shards were found (Supplementary Fig. 8), clearly showing that this layer represents a short accumulation event of volcanic ash and aeolian silt. The chemical composition of glass shards from this tephra layer (Supplementary Fig. 9 and Supplementary Table 3) suggests that this tephra layer is another occurrence of the widespread Campanian Ignimbrite/Y-5 tephra, a crucially important marker horizon found in many locations throughout the Mediterranean, Balkans, and further east^29^,^30^. Originating in the Campi Flegrei volcanic field of Italy and dated elsewhere (^40^Ar/^39^Ar) to 39,280 ± 110 years BP^28^, this is the first report of this tephra find within loess records in southeastern Europe outside the Lower Danube area^29^,^30^. The vegetation cover was probably significantly degraded due to the local impact of the ashfall^29^, enabling the higher availability and dynamics of coarser grain-size particles (Figs 2 and 3). The timing of the Campanian Ignimbrite/Y-5 tephra eruption^27^,^28^,^29^,^30^ also closely matches the timing of Heinrich event 4, which may have resulted in a delay in vegetation recovery and generally colder climatic conditions even several centuries after the eruption. The high sand content indicates unfavorable environmental conditions during the short accumulation period of this layer (ca. 0.70 m) even in the upper part of this layer, where the proxies used for tephra determination (including microscopy) show a limited/suppressed contribution of the volcanic ash (see Supplementary material and Supplementary Fig. 7). It can be concluded that Heinrich event 4 was linked to specially expressed stadial conditions in Southeastern Europe.

It has to be emphasized that studies on glaciers from the Balkans^41^,^42^,^43^ support rather mild last glacial conditions. Those studies showed that maximum glacier expansion in the last 350,000 years occurred during MIS 6^41^,^42^,^43^. Though smaller, the glacier expansions during MIS 10 and MIS 8 were rather similar to those during MIS 6 (especially over the Dinarides^41^). MIS 5-2, however, were characterized by significantly less extensive and smaller glaciers^41^,^42^,^43^. The limited extension of glaciers during the Late Pleistocene, including the period of the last glacial maximum, over the Balkans supports either cold and very dry or, alternatively, generally milder conditions and less expressed glacial-interglacial climate variability. Data from our study support the second scenario with generally milder glacial conditions and/or warmer summer temperatures (higher abundance of fine particles, lower values of the U-ratio and lower L* relative to previous glaciations; Fig. 3 and Supplementary Fig. 12). Similar suggestions have been made from the deep-sea temperature reconstructions based on independent sea level models from the Mediterranean Sea^46^, proposing that the last glacial may have been relatively mild^46^. The milder last glacial conditions were caused by a feedback triggered by the shift from Mediterranean-like climate towards continental climate in MIS 5. An intensification of continental climate conditions over Southeastern Europe shifted the subtropical anticyclone to the south, causing an increase in precipitation during summer, and a decrease of precipitation during winter without significant change in the overall temperature relative to previous interglacials. This shift towards continental climate significantly reduced the extent of glaciers over the Balkans, also during the cold stages after MIS 5. The limited presence of glaciers during the last glacial cycle in the Balkans’ mountains enabled penetration of warm and moist air from the south into the interior of Balkans, leading to the mild glacial conditions in the interior of the Balkans as recorded at Stalać. The Greenland ice-sheet extent during the studied period may had an influence on the stronger continental climate over Central Balkans. A simulation of the Greenland ice-sheet extent during last ~350,000 years^47^,^48^ indicates that only the last interglacial (MIS 5e) was characterized by a large-scale melting of this ice-sheet (Supplementary Fig. 12). This may have established different atmospheric circulations over Europe at that time with a stronger influence of anticyclones from continental Europe (Siberian high) over the Balkans due to the weakening of Greenland high pressure. However, at this stage the inferred pattern is indicative only. If valid, it suggests that the melting of the Greenland ice-sheet can significantly change the future climate in the interior of the Balkans and intensity of the continental climate influence in Middle Danube Basin.

In summary, this study provides the first comprehensive palaeoclimate reconstruction of the Central Balkans for the past ~350,000 years based on the terrestrial sediment proxies. Data show that the Central Balkans experienced different palaeoenvironmental and palaeoclimatic conditions than the Middle and Lower Danube Basins did. The latter were much more strongly influenced by continental climate. Our findings suggest that a sharp climatic transition zone may have existed between these regions. A Mediterranean-like climate prevailed from MIS 10 to MIS 6 in the Central Balkans. However, our data also point to a general trend of progressively weaker Mediterranean climate influence within the entire Southeastern Europe (Fig. 4). An abrupt shift in general climate conditions over the interior of the Balkans to more continental conditions from MIS 5 to present is evident in the data derived from the section, possibly connected to the Greenland ice-sheet retreat during MIS 5e. The prevalence of the continental climate influence over the Central Balkans led to a change in precipitation maxima from winter to warmer periods of the year, resulting in a significant reduction of glaciers in the Dinarides. The absence of large glaciers enabled the penetration of relatively warm and humid air from the south and southwest into the interior Balkans, leading to higher humidity during the last glacial, when compared to previous glaciations.

From a wider perspective, this study has established the spatial and temporal borders of the Mediterranean climate influence and its interaction with the continental climate influence over Southeastern Europe. However, the palaeoclimatic reconstruction of the Middle Danube Basin remains primarily based on environmental magnetic properties of loess-palaeosol sequences, especially for the sequences preserving older records than the last glacial. Specific physical properties and changes in sediment provenance at the Stalać section make it impossible to reconstruct the palaeoclimate based on environmental magnetism (for more details see the Supplementary material). A better understanding of the past climatic relations between these two regions requires more studies based on grain-size and colour analyses from long loess sections in the Middle Danube Basin, but also investigations of the older parts of the Stalać section.

Additionally, our study paves the way for new discussions, studies and hypotheses. For example, the observed different climate conditions in the Central Balkans and Middle Danube Basin indicates a narrow climatic boundary that might explain the role of the Balkan Peninsula as a Quaternary floristic refugium^32^. Such a climatic boundary is thought to have protected the Balkan Peninsula from the adverse climatic conditions in the North. Particularly, enhanced humidity during the last glacial explains why the Balkan Peninsula was an important floristic refugium during this period^32^ and highlights its importance as a European biodiversity hotspot. Our study also has the potential to improve our knowledge concerning the dispersal of anatomically modern humans from Africa to Europe, because the Balkans have been suggested as one of the main migration corridors. It questions the assumption that the appearance of the anatomically modern humans in the Balkans was determined by the unfavorable climate conditions before ~45 ka^49^,^50^,^51^. We emphasize that even though the last glacial was mainly characterized by relatively mild glacial conditions in the Central Balkans, the L1SS1LLL1 layer records the strong impact of the distal ashfall after the Campi Flegrei super-eruption^27^,^28^ and the Heinrich event 4, indicating a drastic shift towards harsh environmental conditions in this area. Although the timing and role of Campanian Ignimbrite/Y-5 ashfall, combined with a sudden climatic change and its interaction between the demise of the Neanderthals and their replacement by anatomically modern humans still remains a matter of hypothesis^29^,^52^, our data suggest a strong impact of the Campanian Ignimbrite and Heinrich event 4 on this region. This supports the assumption that the Campanian Ignimbrite ashfall and a sudden climatic change during the Heinrich event 4 might have played an important role in the demise of Neanderthals, so that the anatomically modern humans could get a permanent hold in Europe only when the Neanderthal populations started to fade.

## Methods

### Grain-size

Subsamples of 0.1–0.3 g fine-earth (<2 mm in diameter) were pre-treated with 0.70 ml of 30% hydrogen peroxide (H_2_O_2_) at 70 °C for 12 hours. This process was repeated until a bleaching of the sediment occurs^53^, but not longer than three days. To keep particles dispersed, the samples were treated with 1.25 ml, 0.1 M sodiumpyrophosphate (Na_4_P_2_O_7_ * 10H_2_O) for 12 h^54^. Particle size characteristics were measured with a LS 13320 Laser Diffraction Particle Size Analyser (Beckman Coulter). To calculate the grain-size distribution the Mie theory was used (Fluid RI: 1.33; Sample RI: 1.55; Imaginary RI: 0.1)^55^,^56^. Clay is represented with particles smaller than 2 μm, fine silt from 2 to 6.2 μm, medium silt from 6.2 to 20 μm, coarse silt from 20 to 63 μm and sand higher than 63 μm^57^.

### Environmental magnetism

The volumetric magnetic susceptibility was measured at frequencies of 300 and 3000 Hz in a static field of 300 mA/m using a Magnon International VSFM. Data were corrected for drift and for the effect of sampling boxes (weak diamagnetism), and normalized to density. Hence, magnetic susceptibility is given as mass specific susceptibility (χ) in m^3^/kg. The frequency dependence was calculated as χ_fd_ = (χ_lf_ − χ_hf_)/χ_lf_*100[%]^6^,^58^.

### Bulk sediment geochemistry

All bulk sediment samples were sieved down to 63 μm and dried at 105 °C for 12 hours. An 8 g quantity of the sieved material was mixed with 2 g Fluxana Cereox wax, homogenized and pressed to a pellet with a pressure of 19.2 MPa for 120 seconds. The measurements were conducted by means of a pre-calibrated method. Samples were analysed for major and trace element abundances with polarization energy dispersive X-ray fluorescence (EDPXRF) using a SpectroXepos.

### Glass shard chemical analysis

The geochemical analyses of glass shards were performed on a sample from layer L1SS1LLL1 with the highest Cl values. The sediment sample was sieved and glass shards were isolated through density separation, mounted in epoxy resin, ground and polished in preparation for microprobe analysis. Measurements were made using single-grain, wavelength-dispersive electron microprobe analysis at the Bayerisches GeoInstitut on a Jeol JXA8200 microprobe employing an accelerating voltage of 15 keV. A 6 nA beam current and defocused beam were used. Order of measured elements (first to last): Na, Si, K, Ca, Fe, Mg, Al, P, Ti, Mn, Cl^−^. Peak counting times were 10 s for Na, 30 s for Si, Al, K, Ca, Fe and Mg, 40 s for Ti and Mn, and 60 s for P. Precision is estimated at <1–6% (2σ) and 10–25% (2σ) for major and minor element concentrations respectively. Analytical settings are presented in Supplementary Table 4.

### Spectrophotometric analysis

A Konica Minolta CM-5 spectrophotometer was used to determine the colour of dried and homogenized sediment samples by detecting the diffused reflected light under standardized observation conditions (2° Standard Observer, Illuminant C). Colour spectra were obtained in the visible range (360 to 740 nm), in 10 nm increments, and these data were then converted into the Munsell colour system and the CIELAB Colour Space (L*a*b*) using the Konica Minolta SpectraMagic NX software. The resultant values indicate the extinction of light on a scale from L* 0 (absolute black) to L* 100 (absolute white), and express colour as chromaticity coordinates on red-green (a*) and blue-yellow (b*) scales.

### Correlative age-model

The age model is based on simple correlation of palaeosols to odd MIS^59^ (S0–MIS 1; L1SS1 and L1SS2–MIS 3, S1–MIS 5, S2–MIS 7 and S3–MIS 9) and loess layers to even MIS (L1LL1–MIS 2, L1LL3–MIS 4, L2–MIS 6, L3–MIS 8 and upper part of L4–late MIS 10), as established for the Middle Danube Basin^25^,^60^,^61^. The age model is confirmed by luminescence dating (Supplementary Fig. 7 and Supplementary Table 5) and volcanic glass shards chemistry (Supplementary Fig. 9 and Supplementary Tables 3 and 4). For more information see section 5 in Supplementary material.

## Additional Information

**How to cite this article**: Obreht, I. *et al*. Tracing the influence of Mediterranean climate on Southeastern Europe during the past 350,000 years. *Sci. Rep.* **6**, 36334; doi: 10.1038/srep36334 (2016).

**Publisher’s note:** Springer Nature remains neutral with regard to jurisdictional claims in published maps and institutional affiliations.

## Supplementary Material

Supplementary Information
