# Supplementary material for: Tracing the influence of Mediterranean climate on Southeastern Europe during the past 350,000 years
Source: Sci Rep. 2016 Nov 8;6:36334. doi: 10.1038/srep36334 (PMC5099887; doi:10.1038/srep36334)
Supplement: Supplementary Information [file srep36334-s1.pdf]

Tracing the influence of Mediterranean climate on Southeastern Europe during the past 350,000 years

Igor Obreht, Christian Zeeden, Ulrich Hambach, Daniel Veres, Slobodan B. Marković, Janina Bösken, Zorica Svirčev, Nikola Bačević, Milivoj B. Gavrilov, Frank Lehmkuhl

1. Sampling strategy

Two separate field campaigns were carried out in June and October, 2013. During the first campaign, a ca. 5.5 m long profile was sampled in the central part of the Stalać section (Supplementary Fig. 1). During the second campaign, four profiles on the south-west side of the brickyard situated at a slope position were sampled from the Stalać section (Supplementary Figs. 2 and 3). It was not possible to sample one continuous section because the brickyard has been mined actively, which had formed terraces. Special attention was paid that all of the sampled profiles were correlated by use of palaeosols and tephra layers; a certain amount of stratigraphic overlap was also included. Before sampling, 10-20 cm of exposed material was removed from all profiles, allowing for continuous, high-resolution, incremental sampling at all five sections. Samples were taken in 5 cm increments, with exception of the fourth profile, where 20 samples from the transition of the lowermost palaeosol to the loess layer above were sampled in 2.5 cm increments. In spring 2014, 13 samples of recent alluvium from the Zapadna Morava (4 samples), Južna Morava (5 samples) and Velika Morava (4 samples) Rivers were collected at 13 different locations (Supplementary Table 1).

Supplementary Table 1. Ni, Cr and magnetic susceptibility values from the Zapadna, Južna and Velika Morava rivers alluvium samples

River	Locality	Cr [ppm]	Ni [ppm]	Magnetic susceptibility [10^{-8} m^3/kg]
Zapadna Morava	Jasina	334.25	165.1	137
Zapadna Morava	Šanac	396.6	272.6	200
Zapadna Morava	Bosnjane	388.65	284.75	129
Zapadna Morava	Maskare	438.4	288.65	165
Velika Morava	Varvarin	241.45	138.95	78.1
Velika Morava	Obrez	301.85	218.25	99.9
Velika Morava	Trešnjevica	193.8	131.25	78.8
Velika Morava	confluence	211.05	115.75	105
Južna Morava	Stalać	145.25	72.25	97.1
Južna Morava	Branjina	135.25	39.5	88.6
Južna Morava	Maletin	157.75	50.65	86.9
Južna Morava	Donji Ljubeš	112.9	49.35	74.6
Južna Morava	confluence	125.1	52.5	106

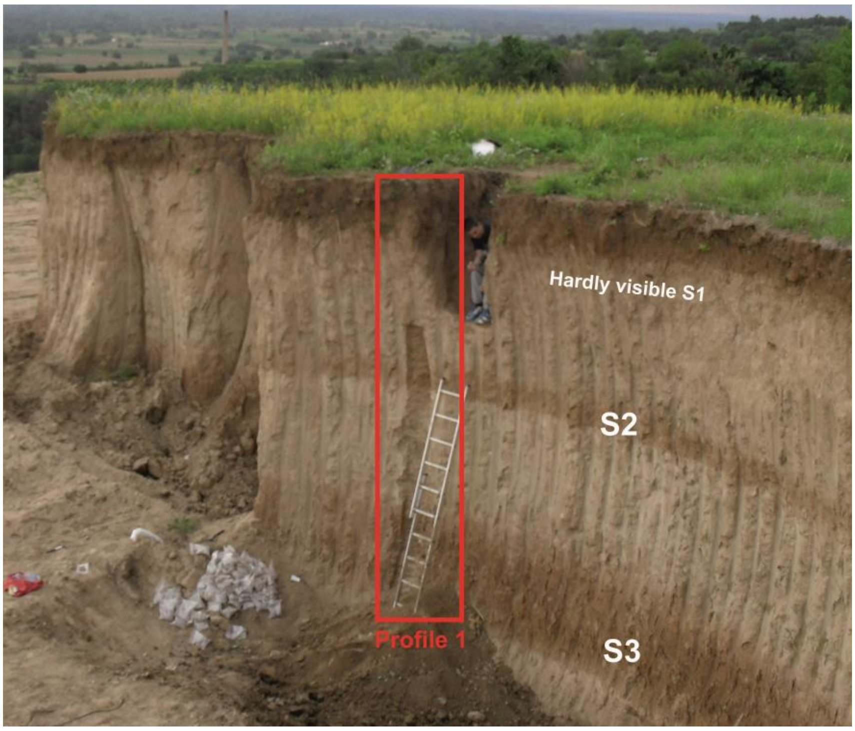

Supplementary Figure 1. Photo of the stratigraphic section sampled (profile 1) during the first field campaign. The profile is marked with a red rectangle. It preserves a record of aeolian sediment over the past ~350,000 years.

2. Stalać section – general features

The studied section (43°40'38.887"N, 21°25'4.18"E) is exposed in an active brickyard at the top of the Južna (South) Morava River terrace. The section is located about 3 km from the confluence of the Južna Morava and the Zapadna (West) Morava Rivers, forming the Velika (Great) Morava River (Fig. 1 in the original manuscript). For the period from 1981 to 2010, the mean annual temperature and mean annual precipitation of the nearby climate station at Kruševac were 11.4°C (with high inter-annual variability) and 628 mm (with the main precipitation maximum during June, and a second maximum during November)[1]. The Stalać section is exposed in a wall that has a north-east to south-west orientation. The north-eastern and central part of the section show several loess and palaeosol layers, preserving proxy data for glacial and interglacial cycles. Six well developed, brown-reddish pedocomplexes, one hardly visible weak brown-grey palaeosol, and the modern soil, intercalated by loess layers, are exposed here. The south-western side of the exposure is developed on a slope and preserves the sediment formed from the last brown-red palaeosol to the modern soil. This younger sediment is preserved in much higher resolution. All palaeosols exposed at the south-west profile are inclined, following the slope exposure; they become thicker downslope. Palaeosols exposed on the slope exposure are bifurcating: the older brown-red palaeosol splits into two palaeosols and the younger (brown-grey palaeosol) splits into three. Supplementary Fig. 2 shows the correlation of the central part and south-western part of this section and Supplementary Fig. 3 shows the spatial correlation of the profiles. For this study, the upper part of the central profile (containing two brown-red palaeosols, a weak brown-grey palaeosol, the modern soil and the various intercalated loesses) and four profiles, spanning all the studied stratigraphic units from the south-western part.

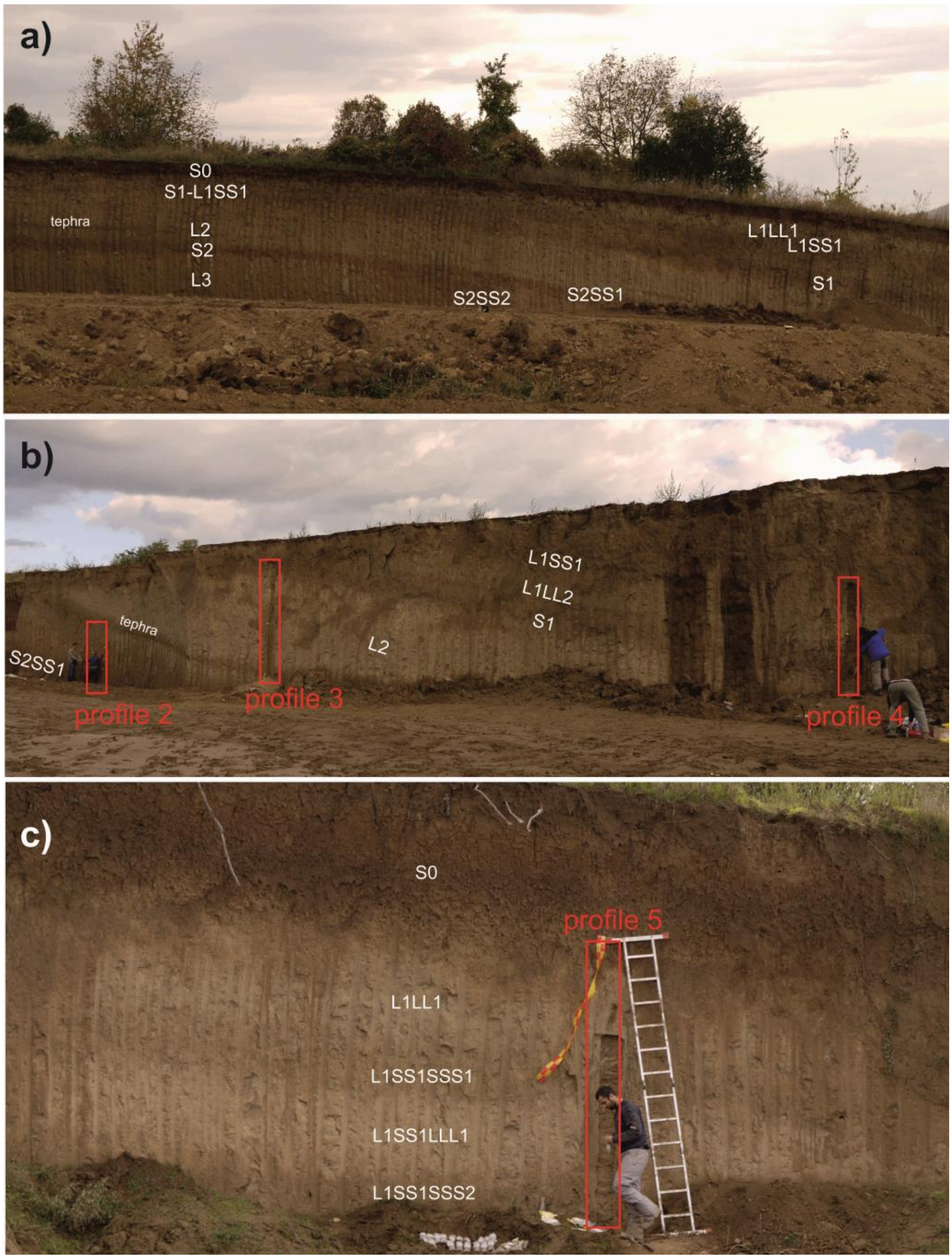

Supplementary Figure 2. Photos of the stratigraphic sections, with labels to aid in correlation. Photos are of the central profile a) and the south-western part sampled during the second field campaign b) - profile 2-4 and c) – profile 5. Sampled areas of the profiles are marked with red rectangles. The tephra layer found in profiles 1, 2 and 3 corresponds to the same layer.

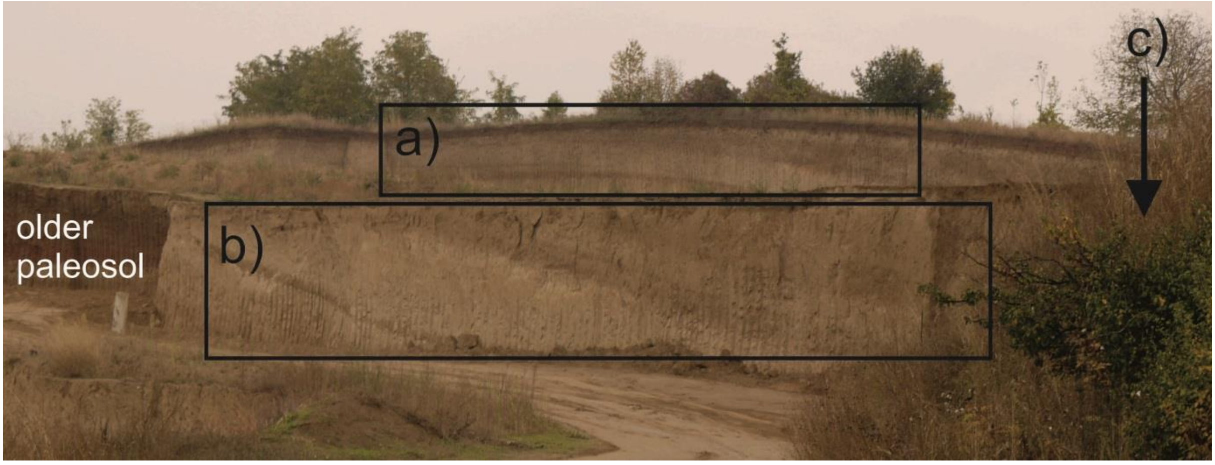

Supplementary Figure 3. Correlation of the profiles: a) profile 1 (as in Supplementary Fig. 2), b) profiles 2-4 (as in Supplementary Fig. 2), c) indication of profile 5. Due to semicircular excavation shape of this site, profile 5 is not shown here but on Supplementary Fig. 2.

3. Stratigraphy of the studied profiles

Profile 1 is 5.55 m long. Thicknesses and depths are reported from the base of the section upward, with zero being the bottom of the section. At the base, 0.4 m of loess (unit L4) is exposed. Above it is a strongly developed, brown-red Cambisol with strongly expressed vertic characteristics (S3); it has a total thickness of 1.25 m (from 0.4 to 1.65 m). The lowermost 0.4 m of the palaeosol has a brown-reddish B horizon. From 0.8 to 1.3 m, a strongly developed, brown-red B horizon is exposed and it is darker n colour than layer below. At the top of this layer, secondary carbonate features are developed. Both horizons have a blocky structure typical for vertic horizons. From 1.3 to 1.65 m, a red-brown Ah horizon with a granular structure is preserved. Above this palaeosol pedocomplex, from 1.65 to 3 m, is a layer of light yellow loess (L3). At 2.6 m, one krotovina was observed but not sampled. From 3 to 3.45 m, another Cambisol with vertic characteristics is exposed (S2), although it is less strongly developed than the

palaeosol below. Typical porous loess (L2) is exposed this soil, from 3.45 to 4.4 m. This loess layer is in turn overlain by a very weak, grey soil horizon with a thickness of ca. 0.25 m (covering S1 - L1SS1). From 4.65 to 5 m, the uppermost and youngest loess layer is found (L1LL1). Within the top of this loess (from 5.0 to 5.55 m) the modern soil (S0) is exposed, with a 0.1 m thick Ah horizon and with granular structure at the bottom, an Ah (mollic) horizon with granular structure in the middle and an Ap horizon on top (strongly affected by human activities showing debris of bricks).

Profile 2 presents the oldest (stratigraphically lowest) part of the profiles on the slope. At the bottom the first 0.2 m were built up by loess. A well-developed reddish palaeosol (0.2 m – 1 m) is formed above the S2LL2 loess layer and present the S2SS2 interstadial soil. On top of this soil 0.3 m of loess were deposited covering a weakly developed 0.15 m thick deep red palaeosol (S2SS1). Above 1.45 m, pale yellow loess (L2) is exposed showing some black organic spots (1.45 – 2.9 m). The grey and coarse tephra layer is situated on the top of profile (~2.9 – 2.95 m).

The bottom of profile 3 begins ca. 0.2 m below the tephra layer. The tephra itself is ca. 0.05 m thick. Above follows a thick loess layer (3.85 m) containing some carbonate concretions in the lower part (L2). The loess layer is intercalated by weak organic layers at ca. 3.05-3.15 m and 3.50-3.60 m. On top, a chernozem-like pedocomplex (S1) with calcareous root channels was formed (4.1 – 4.45).

Profile 4 starts in loess unit L2 (0 – 0.5 m) and continues to the ca. 0.85 m thick palaeosol S1. Above the palaeosol, a 1.30 m thick loess layer is exposed. From 2.65 m to the top of the sampled profile (3.05 m) a palaeosol is developed.

The bottom of profile 5 corresponds to the upper palaeosol from profile 4. Here, this palaeosol has a thickness of 1.00 m. It is followed by a darker loess layer (1.00 – 1.85 m).

Above, another palaeosol horizon (1.85 – 2.65 m) is developed. A brown loess layer is exposed from 2.65 – 3.7 m. On top, the modern soil is exposed. The top of the modern soil is heavily influenced by human activities, thus only 30 cm of its lowermost part were sampled.

4. Composite profile splicing

Major parts of the five separately sampled profiles were spliced to obtain a composite profile from the Stalać section. The stratigraphy of profiles 2 to 5 was evident from the inclined loess and palaeosol units on one brickyard terrace; sampling was performed in order to have some overlap between these sub-profiles. Supplementary Fig. 4 shows the overlap of some physical and chemical property data used for the splice (magnetic susceptibility, U-ratio, L*, a*, CaO and Cl). Sampling the inclined profiles 2-5 may potentially entail the sedimentary succession with its real – vertical – sediment accumulation. When assuming non-vertical accumulation, but accumulation perpendicular to the slopes, the sampling depth is overestimating the actual sedimentation thickness, and might somewhat mix non-syndepositional sediment. Due to the high sampling resolution and the clear autocorrelation of the data we do not regard this as an issue, but it is important for the understanding of the sedimentary succession.

Profile 1 covers the past ~350,000 year. However, the past 191 ka (S2-S0) is preserved in very low resolution and possibly in discontinuous sedimentation and/or preservation. Therefore, we used only the lower part of profile 1 from upper part of L4 to end of S2 for the splicing. Profile 2 covers the period of formation of the upper part of S2 and the lower part of L2. Since this profile does not cover the whole S2, it is very complicated to splice the upper part of S2 palaeosol (S2SS1) from profile 1 to whole S2

palaeosol from profile 1 because they exhibit different temporal resolutions. Thus, the profile was spliced at the end of S2 at profile 1 and on the beginning of L2 at profile 2. In general, the correlation of profiles 1 and 2 is not straight forward since those profiles formed in different geomorphological settings. It has to be stressed that profile 2 to 5 were deposited on the slope and thus may be formed under different depositional regimes. Thus, the transition from profile 1 to profile 2 was treated with special attention while interpreting the data. Despite differences in some of the bulk data (e.g. colour), we argue that general palaeoclimate signals and trends can be clearly extracted, since similar patterns are observed on the L2 layers of both profiles. However, it has to be noted that it is not possible to directly compare the sedimentation rates from profile 1 to profiles 2-5. Therefore, this study does not focus on the sedimentation rates, especially because sedimentation rates may not be representative for sediments deposited on the slope.

Splicing of profiles 2 and 3 was straight forward. We connected the profiles via the tephra layer (Supplementary Fig. 4).

Splicing of profiles 3 and 4 was a bit more complicated. The palaeosol pedocomplexes developed on the slope may have overprinted the sediment below in a different way. However, it seems that this effect is negligible, since most of the proxies show very similar values on both profiles just at the transition from loess to the S1 palaeosol (Supplementary Fig. 4).

More challenging is the splicing of profiles 4 and 5. Profile 4 did not reach the end of the upper L1SS2 palaeosol while profile 5 started just at the beginning of the same palaeosol unit with no overlap to the loess layer below. The lower limits of the palaeosols do not have a fit as good as between profiles 3 and 4. Thus, the splicing was not performed at

the bottom of the palaeosol but within the palaeosol where most of the proxies showed similar values (ca. 0.25 m below the end of profile 4 and 0.2 m above the beginning of profile 5; Fig 4). It may be possible that a few centimeters of the sediment are present twice or are missing, but generally data variation is low. Thus, we argue that this correlation does not suffer from major issues. The final composite profile is presented at Figs. 5 and 6.

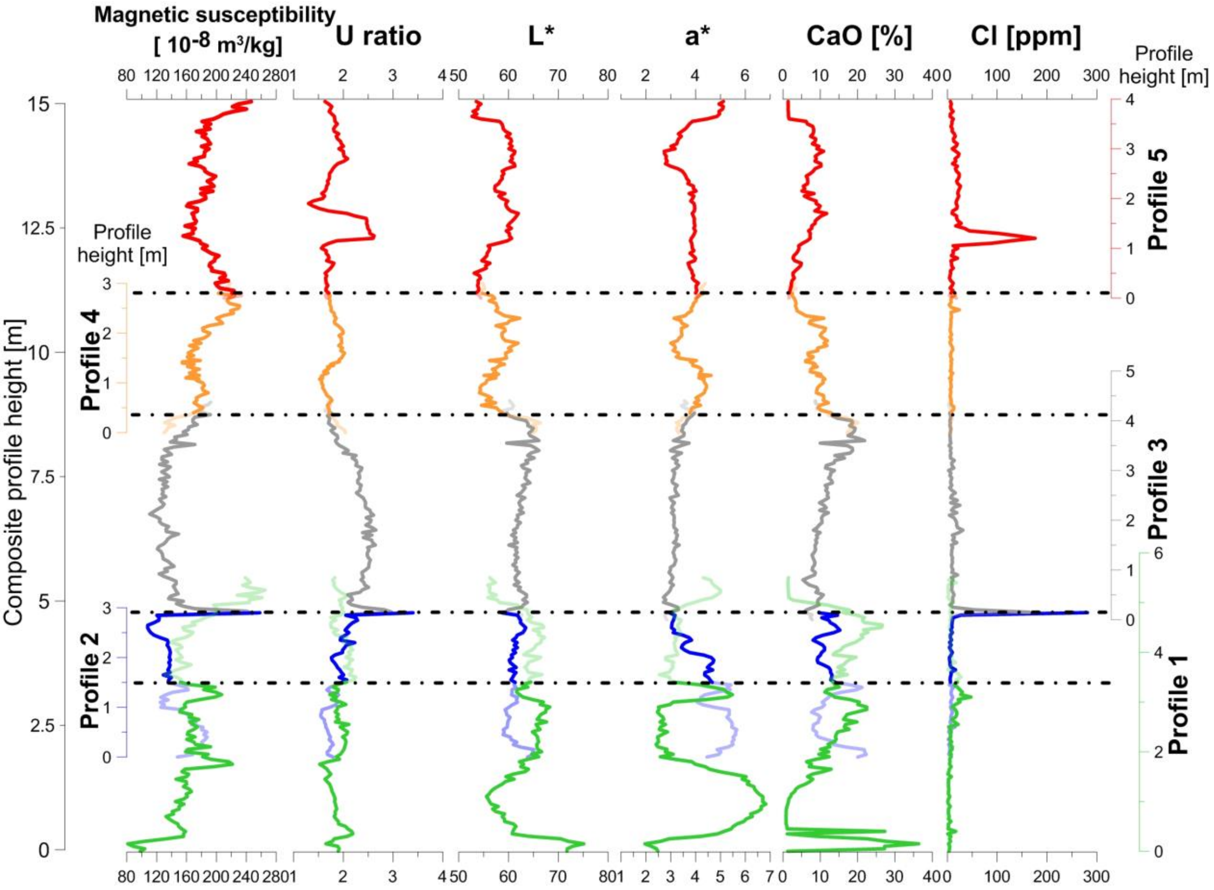

Supplementary Figure 4. Splicing of profiles 1-5. Dashed black lines present the splicing points. Light lines of records present the part of profiles that was not used in the composite profile. Note that the upper part of profile 1 is compressed, revealing a different sedimentation rate. The difference in pattern between profiles 1 and 2 arises from different sedimentation rates; tephra occurrence provides a certain marker in L2 facilitating correlation between profiles 1 and 2.

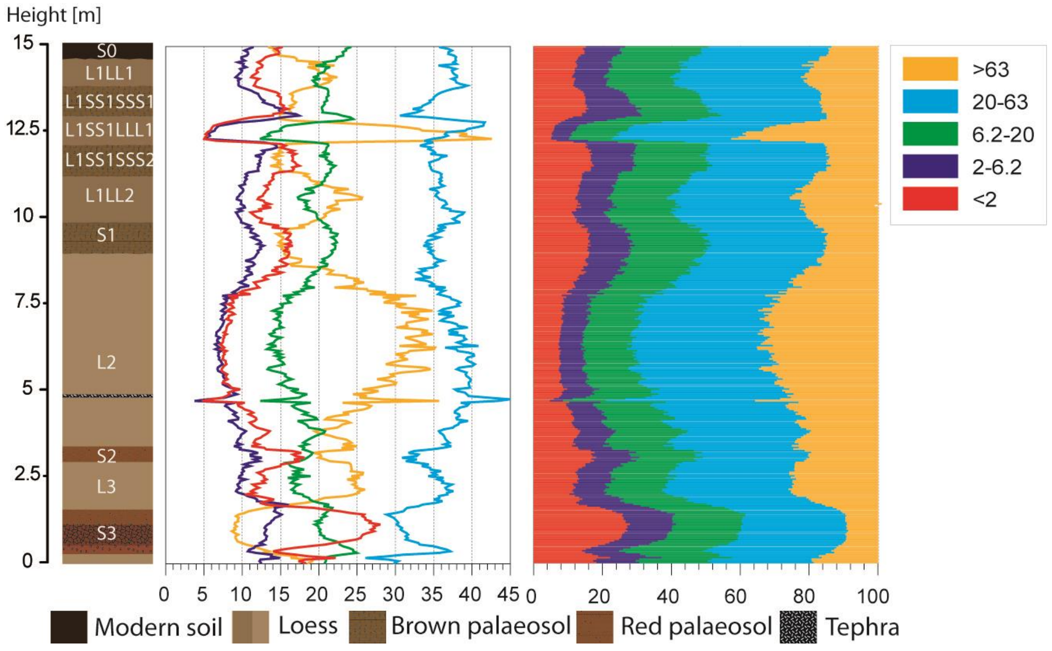

Supplementary Figure 5. Grain-size distribution of each particle size class and cumulative distribution from all grain-size classes (each colour in the right box represent a corresponding class in μm) of the composite profile.

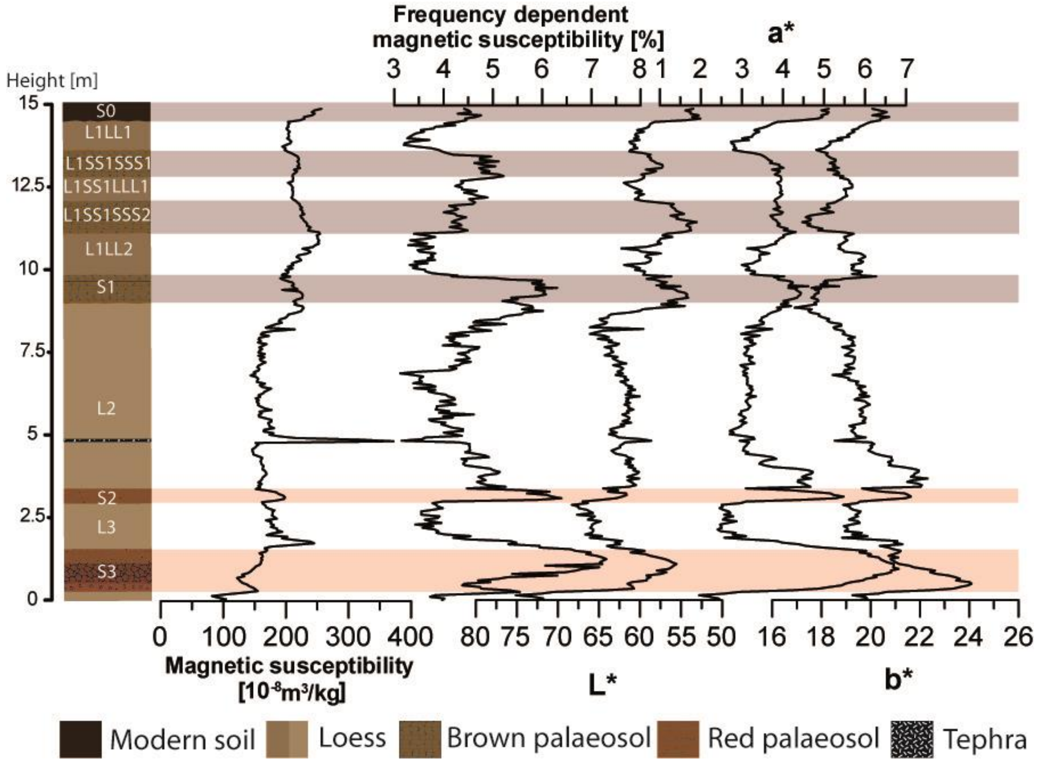

Supplementary Figure 6. Magnetic susceptibility, frequency dependent magnetic susceptibility, L*, a* and b* values related to pedostratigraphy of the composite profile.

5. Chronology and age model

Geochronology of loess-palaeosol sequences in the Middle Danube Basin has generally been established mainly by ^{14}C and luminescence dating for the younger time periods[2–5]. Nevertheless, many loess-palaeosol sequences spanning several glacial cycles cannot be dated by these methods. Some sections were dated by correlation of the magnetic susceptibility (χ) signal to oxygen isotope records of marine benthic foraminifera or orbital variations[6–11]. Such correlative age models may not be accurate on the scale of few thousand years, but in the absence of other dating techniques this method is generally accepted to provide reliable timescales. Since the χ at the Stalać section is strongly biased by provenance change, we applied correlation of odd Marine Isotope Stages (MIS) to phases of soil formation. The first chronostratigraphy of Stalać based on correlation of the soils to interglacials and loess to glacials was established more than a decade ago[12]. However, our investigations including tephra correlations suggest that this may be more challenging and that the previous chronology is not correct. This fundamentally changes our understanding of this region, suggesting that the previously proposed mutual climate connections between Central Balkans and Middle Danube Basin[12] have to be reconsidered.

In general, the stratigraphic framework of loess and the labeling of loess-palaeosol layers in the Middle Danube Basin is simple because the sequences were formed by relatively continuous deposition of aeolian dust[13,14] with only rare exceptions[15,16]. The labeling of stratigraphic units follows the established scheme for the European loess-palaeosol sequences presented by Marković et al.[14], using 'L' for loess units and 'S' for palaeosol units, while the ordinal numbers indicate the order of increasing age. Although the stratigraphic framework seems simple, the past climate characteristics of the central

Balkans are not well understood. Thus, the determination of secure pedostratigraphic markers for simple stratigraphic linking of profiles is more complicated. For example, the correlation of visible layers is becoming challenging for the south-western section of the studied outcrop since the palaeosols there are splitting downslope.

One of approaches for obtaining age models used in Southeastern Europe is orbital tuning[6,9]. Basarin et al.[6] orbitally tuned a loess record in the Middle Danube Basin. Their record spans almost the last million years, which allows for the determination of specific spectral (frequency) properties in depth and time. Our record spans ca. 350 ka, which is at the very low end of a useful length for orbital tuning. This is especially the case as the sedimentological patterns seem to be dominated by global and regional climate changes on glacial-interglacial level, and do not dominantly correspond to one of the Milankovitch frequencies (precession, obliquity, eccentricity). Therefore we refrain from a tuning approach here, but use a correlation to other proxy records to establish an age model instead.

Two tephra layers were identified in studied profiles at the Stalać section. The lower tephra likely corresponds to the L2 tephra observed in many loess-palaeosol sequences in the Middle Danube Basin[14,17,18]. Although this tephra layer is clearly visible in the field, it underwent significant alteration and glass shards are not preserved. The upper tephra was not visible in the field but the analyzed data clearly pointed to a tephra layer (especially the grain size trends (Supplementary Fig. 5), high Cl concentration (Supplementary Fig. 7) and microscopically observed volcanic glass shards (Supplementary Fig. 8)). The results from microprobe geochemical analyses of glass shards (Supplementary Fig. 9 and Supplementary Table 3) clearly relate this layer to the Campanian Ignimbrite/Y-5 tephra (~39 ka[19–21]) coming from the Campi Flegrei caldera

in Italy, and identified so far in many locations throughout the Mediterranean Sea, in loess archives in the Lower Danube[19–21], and throughout records in the Balkans[22,23]. This volcanic ash layer has not been observed in the χ trend (albeit recorded in the Cl data), in contrast to the L2 tephra that is clearly visible in the data (Supplementary Fig. 6). The reason for this lies in the peralkaline trachytic composition of the Campanian Ignimbrite/Y-5 tephra. The χ of the Campanian Ignimbrite/Y-5 tephra does not exceed ~$200*10^{-8}$ m^3/kg[20], thus having already lower values than the bulk loess sediment at Stalać. Since this tephra layer is not visible by naked eye, it is very likely it appears as a crypto tephra mixed up with some degree with aeolian silt. Beside these two tephra layers, below the studied profile and thus beyond the frame of this study, another potential tephra layer was observed in the middle of the L4 unit. This tephra may correspond to the Bag tephra observed in the Middle Danube Basin[14,17]. This provides additional (albeit tentative) evidence that supports the established chronology.

Supplementary Fig. 7 presents the composite profile with tephra proxies (Cl and χ) correlated to the age model. The glass-shard chemical composition assigns the upper volcanic ash bed to the Campanian Ignimbrite/Y-5 tephra, with an age of ~39 ka[19–21], while the occurrence of the L2 tephra is tentatively related to the MIS 6. The age model is based on a simple correlation of palaeosols to odd MIS[24] (S0 – MIS 1; L1SS1 and L1SS2 – MIS 3, S1 – MIS 5, S2 – MIS 7 and S3 – MIS 9) and loess layers to even MIS (L1LL1 – MIS 2, L1LL3 – MIS 4, L2 – MIS 6, L3 – MIS 8 and upper part of L4 – late MIS 10), as established for the Middle Danube Basin[6,7,9,14]. An exception is layer L1SS1LLL1 that is attributed (at least in its lower part) to a mixture of Campanian Ignimbrite/Y-5 ash and coarse dust particles (Supplementary Fig. 7), and it is used as an additional tie point (Supplementary Table 2).

To test the age-model additional evidence (albeit preliminary) from luminescence dating[25] is also briefly discussed. For equivalent doses (De) determination, fine-grained (4-11μm) polymineral samples were measured in a Risø TL/OSL DA 20 reader at the Cologne Luminescence Lab. The post infrared infrared stimulated luminescence (pIRIR) protocol by Thiel et al.[26] and the central age model[27] were used. Prior to IR stimulation temperature tests and dose recovery tests were conducted satisfactorily. Dose rates were determined through the measurement of radionuclide concentrations in a high-purity germanium gamma-ray spectrometer, corrected by conversion and attenuation factors of Guerin et al.[28], Brennan[29], Mejdahl[30], and the measured water content. A potassium content of 12.5±0.5% was assumed[31]. Alpha efficiencies of 0.13±0.01 for C-L3780 and 0.11±0.01 for C-L3784 and C-L3786 were employed. The preliminary luminescence ages are presented in Supplementary Fig. 7 and Supplementary Table 5 (the reader is referred to Bösken et al.[25] for further details) and all ages are in very good agreement with the proposed age model. Nevertheless, the preliminary luminescence ages[25] are in low resolution, and thus cannot be employed at this stage in constraining the millennial-scale climate oscillations, but they incontestably indicate validity of the age-model discussed.

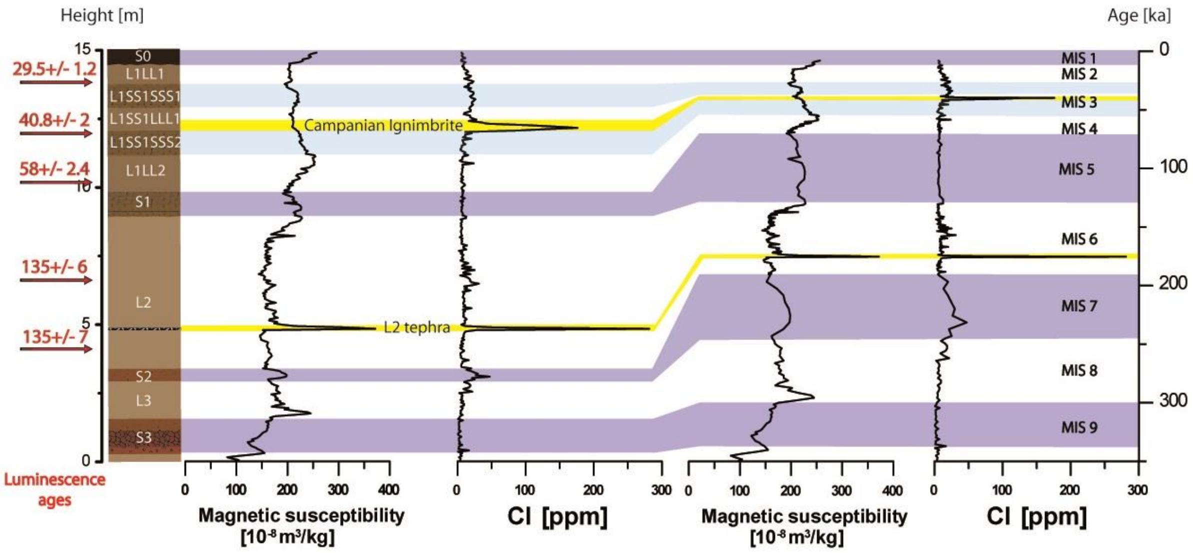

Supplementary Figure 7. Correlation of height scale and the resulting age assignment of different units at Stalać. Bulk sediment geochemical proxy Cl and magnetic susceptibility respectively, provided useful proxies for the identification of tephra layers. On the left, preliminary luminescence ages[25] are presented.

Supplementary Table 2. Tuning points of Stalać age model to Marine Isotope Stages based on LR04 stack[24]

Tuning point	Height (m)	Age (ka)
Beggining of profile	0	350
L4/S3 transition	0.4	337
S3/L3 transition	1.65	300
L3/S2 transition	3	243
S2/L1 transition	3.45	191
L1/S1 transition	8.9	130
S1/L1LL3 transition	9.75	71
L1LL3/L1SS2 transition	11.05	57
L1SS2/L1LL2 transition	12.15	40
L1LL2/L1SS1 transition	12.85	38
L1SS1/L1LL1 transition	13.7	29
L1LL1/S0 transition	14.65	11
End of profile	15	8

Supplementary Table 3. Major oxide geochemical results from microprobe analyses of glass shards from upper crypto tephra layer (the Campanian Ignimbrite/Y-5) at the Stalać section. Data are presented as raw values. Analytical settings used for determining the glass-shard major oxides composition is presented at the Supplementary Table 4.

Location	SiO_2	TiO_2	Al_2O_3	FeO	MnO	MgO	CaO	Na_2O	K_2O	P_2O_5	Cl^-	Total
Stalać	58.79	0.35	18.09	2.77	0.22	0.35	1.60	5.63	6.56	0.05	0.65	95.06
	59.31	0.40	18.10	2.88	0.24	0.31	1.69	5.75	7.00	0.03	0.71	96.42
	60.12	0.43	18.45	2.81	0.23	0.33	1.67	6.14	6.73	0.01	0.72	97.64
	58.08	0.33	17.50	3.25	0.08	0.66	2.35	2.88	8.88	0.19	0.30	94.50
	60.12	0.42	18.48	2.83	0.23	0.30	1.62	6.35	6.81	0.04	0.73	97.92
	59.98	0.42	18.47	2.86	0.23	0.30	1.64	6.49	6.69	0.01	0.69	97.77
	59.33	0.42	18.06	2.84	0.21	0.32	1.66	6.17	6.53	0.05	0.76	96.35
	60.59	0.41	18.61	2.95	0.21	0.35	1.68	6.46	6.87	0.04	0.74	98.91
	59.35	0.42	18.24	2.79	0.22	0.33	1.61	6.08	6.88	0.05	0.70	96.67
	59.47	0.39	18.25	2.79	0.19	0.31	1.64	6.30	6.52	0.04	0.74	96.64
	58.70	0.41	17.93	2.80	0.25	0.35	1.59	6.28	6.43	0.05	0.74	95.52
	58.68	0.39	17.99	2.77	0.20	0.33	1.74	5.85	6.93	0.07	0.73	95.67
	58.13	0.43	18.08	2.79	0.22	0.32	1.77	5.55	7.00	0.05	0.76	95.10
	59.47	0.41	18.20	2.87	0.21	0.32	1.57	6.29	6.66	0.03	0.67	96.70

58.87	0.38	17.86	3.33	0.11	0.71	2.46	2.96	9.28	0.18	0.28	96.43
58.60	0.44	18.30	2.71	0.21	0.30	1.63	5.84	6.53	0.06	0.74	95.35
60.65	0.46	18.53	3.04	0.23	0.31	1.60	6.51	6.91	0.01	0.68	98.94
58.96	0.42	18.59	2.77	0.22	0.30	1.70	5.84	6.60	0.04	0.75	96.18
58.41	0.38	17.88	2.84	0.19	0.29	1.61	5.74	6.60	0.05	0.72	94.70
58.74	0.38	18.20	2.83	0.21	0.32	1.76	5.89	6.97	0.04	0.75	96.10
59.58	0.39	18.18	2.83	0.25	0.31	1.69	5.99	6.95	0.03	0.78	96.99
60.72	0.42	18.49	2.94	0.22	0.32	1.64	6.28	7.24	0.00	0.74	99.02
59.87	0.39	18.50	3.01	0.22	0.34	1.76	6.47	6.85	0.03	0.78	98.21
58.45	0.36	17.38	3.05	0.09	0.68	2.29	3.29	9.12	0.10	0.32	95.13
59.91	0.36	17.85	3.14	0.12	0.65	2.34	3.27	9.13	0.14	0.31	97.23
59.24	0.39	18.47	2.90	0.25	0.33	1.69	5.52	7.04	0.02	0.67	96.52
58.27	0.37	17.90	3.16	0.09	0.72	2.45	3.40	8.59	0.09	0.30	95.34
58.78	0.44	17.96	3.29	0.12	0.72	2.51	3.37	9.21	0.13	0.30	96.83
58.40	0.35	17.82	3.18	0.11	0.71	2.36	2.91	9.03	0.13	0.31	95.32
58.18	0.36	17.71	3.33	0.04	0.72	2.47	2.92	9.63	0.17	0.27	95.81
57.76	0.42	17.69	2.86	0.24	0.33	1.72	5.86	6.52	0.02	0.78	94.21
58.52	0.44	18.32	2.80	0.21	0.34	1.63	5.64	6.74	0.07	0.64	95.34

	60.23	0.46	18.37	2.96	0.20	0.37	1.66	6.19	6.91	0.08	0.74	98.17
	60.01	0.47	18.23	2.90	0.20	0.33	1.65	6.45	6.71	0.01	0.78	97.75
	58.48	0.44	18.23	2.78	0.28	0.33	1.72	5.97	6.81	0.03	0.69	95.77
	60.42	0.42	18.30	2.81	0.20	0.31	1.57	6.20	6.83	0.09	0.68	97.83
	60.80	0.39	18.38	2.92	0.22	0.35	1.64	6.28	6.96	0.02	0.67	98.63
	59.79	0.39	18.38	2.78	0.21	0.32	1.59	6.25	6.77	0.05	0.68	97.21
	58.77	0.46	17.75	2.78	0.22	0.32	1.74	4.99	6.83	0.13	0.72	94.71
	59.54	0.39	18.27	2.87	0.23	0.35	1.69	6.44	6.55	0.03	0.77	97.13
	58.13	0.38	17.94	3.30	0.08	0.73	2.55	2.94	9.33	0.18	0.30	95.87
	59.61	0.43	18.02	2.92	0.27	0.34	1.67	6.35	7.24	0.05	0.75	97.65
	60.24	0.40	18.30	2.98	0.21	0.33	1.69	6.32	6.96	0.04	0.75	98.23
	58.94	0.32	18.08	3.32	0.13	0.73	2.51	3.03	9.47	0.19	0.30	97.02
Average	*59.25*	*0.40*	*18.14*	*2.94*	*0.19*	*0.41*	*1.84*	*5.39*	*7.34*	*0.07*	*0.63*	*96.60*
St. Dev.	*0.83*	*0.04*	*0.29*	*0.18*	*0.06*	*0.16*	*0.33*	*1.30*	*1.03*	*0.05*	*0.18*	*1.30*

Supplementary Table 4. Analytical settings used for determining the glass-shard major oxides composition at Bayerisches GeoInstitut, Bayreuth University. Order of measuring elements (first to last): Na, Si, K, Ca, Fe, Mg, Al, P, Ti, Mn, Cl^-.

**Element**	**Standard (Standard-Block)**	**Measuring time (peak/bkgr.)**
Si /Kα	C-Forsterite (Cameca Block5)	30/15
Mn /Kα<br>Ti /Kα	C-MnTiO3 (Cameca Oxides (2))	40/20<br>40/20
Al /Kα	C-Spinel (Cameca Block5)	30/15
Fe /Kα	Fe-GP40 (GP40)	30/15
Mg /Kα	C-Enstatite (Cameca Block5)	30/15
Ca /Kα	C-Wollastonite (Cameca Oxides (2))	30/15
K /Kα	C-Orthoclase (Cameca Oxides (2))	30/15
Na /Kα	C-Albite (Cameca Oxides (2))	10/5
P /Kα	C-Apatite (Cameca Oxides (2))	60/30
Cl^- /Kα	C-Vanadinite (Cameca Oxides (2))	30/15

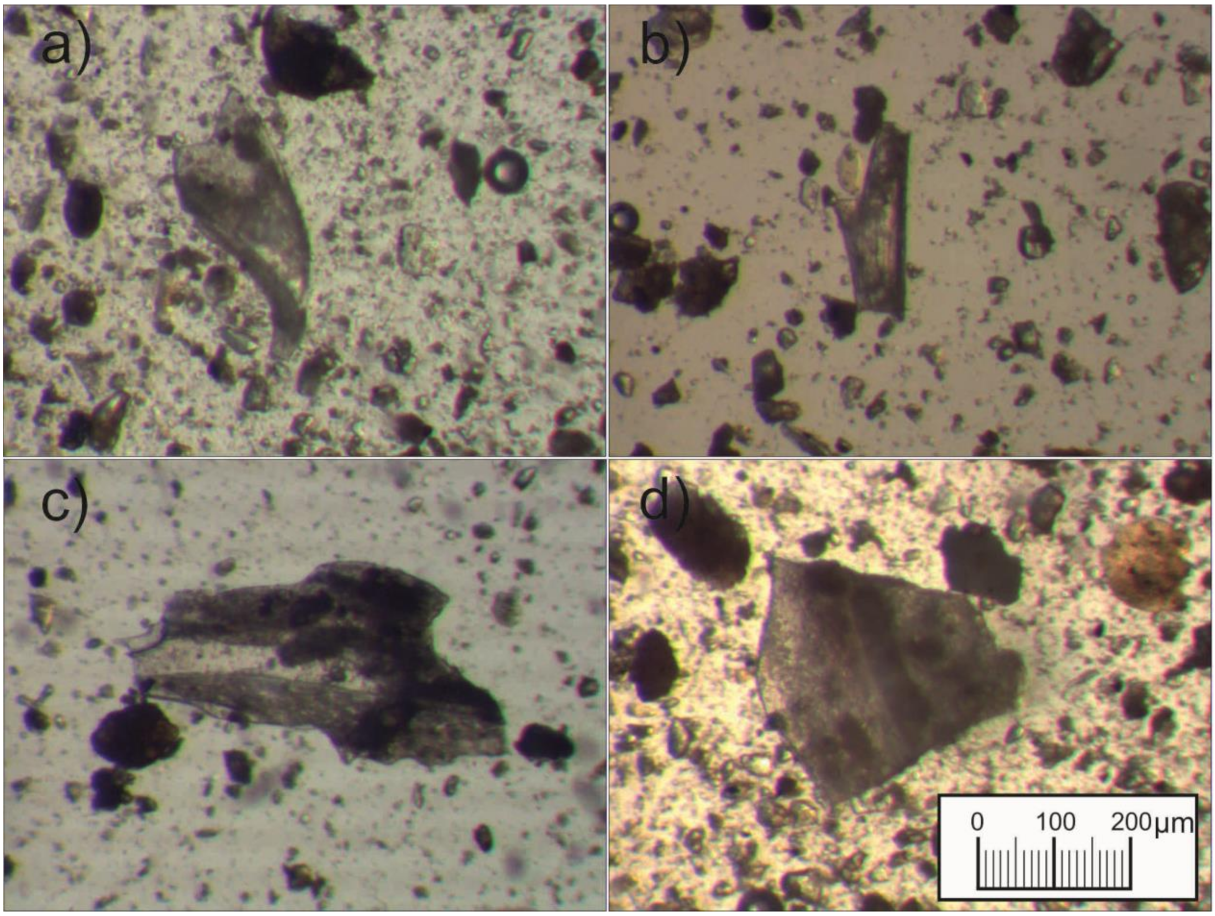

Supplementary Figure 8. Microscopical photos of volcanic glass shards from cryptotephra L1SS1LLL1 layer. The images were taken with magnification factor 50. The sediment was suspended in water and viewed in transmitted light.

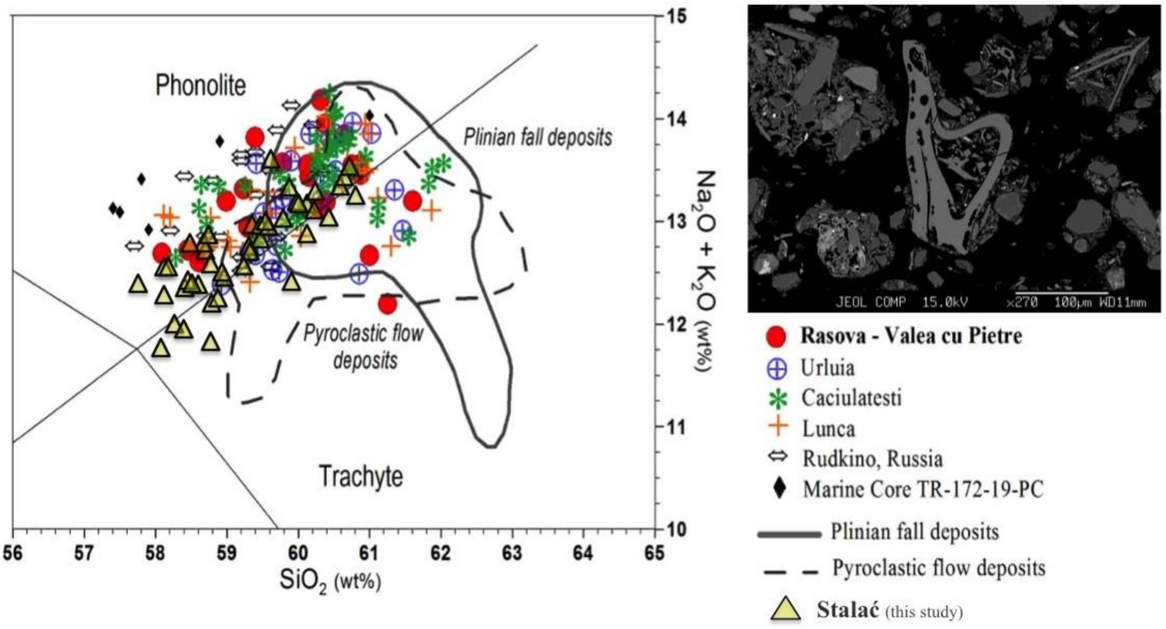

Supplementary Figure 9. Total alkali - Silica diagram (modified after Anechitei-Deacu et al.[32]) showing the geochemical correlation between the Campanian Ignimbrite tephra identified at Stalać and other regional occurrences, including proximal pyroclastic flow[33] and plinian fall deposits in Italy[34], as well distal fine ash occurrences within Mediterranean marine records[35] and in the Russian loess[35], the terrestrial sequence at Caciulatesti in southern Romania[21], and Urluia[20] and Rasova – Valea cu Pietre[32] loess/paleosol sequences in the Lower Danube loess. Inset: scanning electron microscope (SEM) image of the Campanian Ignimbrite glass shards from Stalać.

Supplementary Table 5. Summary of the De, dose rate and resulting pIRIR age data[25]. Water content was obtained in the laboratory. Equivalent doses (De) are shown as result of central age model[27]. Standard errors are indicated. Ages are expressed with a 1-sigma error range.

Sample name	Lab code	Grain-size (μm)	Water content (%)	Total dose rate (Gy/ka)	D_e (Gy) CAM/COM	Age (ka)
St 3	C-L3780	4-11	11.8 ± 5.9	4.07 ± 0.2	648±35	135±7
St 7	C-L3784	4-11	8.6 ± 4.3	4.06 ± 0.2	621±32	135±6
St 9	C-L3786	4-11	8.0 ± 4.0	3.71 ± 0.2	245±13	58±2.4
St 10	C-L3787	4-11	10.7 ± 5.4	3.97 ± 0.2	192±10	40.8±2
St 11	C-L3788	4-11	7.8 ± 3.9	4.26 ± 0.2	149±8	29.5±1.2

6. Principle of geochemical tracing palaeo-river dranaige network over the Central Balkan

The grain-size records generally show remarkably coarser grain distributions (Supplementary Fig. 5) compared to the other sections in Southeastern Europe[13,16,18,36,37] covering the same time interval (Fig. 3 in the original manuscript). This indicates that the Stalać section reflects mainly an enhanced dust contribution from proximal sources. The density distribution curve indicates primary aeoilan deposition (Supplementary Fig. 10). The possible source areas for the Stalać section are the valleys of Južna Morava, Zapadna and Velika Morava Rivers (Fig. 1 in the original manuscript). These rivers present the main drainage network of the Central Balkan, and Stalać is located at the confluence of these rivers. The geology in the catchment of these rivers is characterized by metamorphic and igneous rocks providing minerals with χ about one order of magnitude higher than in loess deposits along the Danube and in China[38]. At the Stalać section, χ is not closely following the pedostratigraphy (Supplementary Fig. 6), as in most other sections in the Middle and Lower Danube Basins[7,8,14,39]. Supplementary Table 1 presents the χ of sediments from the possible source areas. We argue that at

Stalać changes in χ are mainly determined by changes in source area rather than by pedogenic processes, and can therefore not be considered as a reliable palaeoclimate proxy. Also changing wind intensity[40,41] may play a role, though an unclear relation of χ and other paleoenvironmental proxies (specifically the Ni and Cr content, and the L* and a*) do not support a dominant wind or provenance component. The χ signal here is probably resulting from available source material, wind intensity and also pedogenic processes including carbonate presence/absence. The Zapadna Morava alluvium has higher χ values compared to the Južna Morava (Supplementary Table 1), and an increase in particle contribution from the Zapadna Morava would be reflected as an increase in χ, and vice versa. The middle part of the Zapadna Morava River catchment is built from ultramafic rocks, which have a high contribution of Ni and Cr[42] and contain a high amount of magnetic iron minerals. Because Ni and Cr are considered more reliable proxies for provenance here than the χ, geochemical proxy data is used to discuss provenance. Elevated concentrations of Ni and Cr are found in the Zapadna Morava alluvium downstream from the ultramafic rock band (Supplementary Table 1). Consistence of Ni, Cr and χ changes within the section present a clear hint at periodic shifts in the domination of particles originating from the alluvium carried by the Zapadna and Južna Morava. Generally, lower values of Ni and Cr are demonstrating the predomination of the Južna Morava River, while higher values of Ni and Cr indicate domination of Zapadna Morava River particles.

Generally, changes in aeolian sediment provenance area are related to changes in wind directions, wind vigor and/or source area[16,37]. However, the main source area is in the proximity of the Stalać section and changes in the wind direction will not change the main source area greatly. Since the provenance changes inferred from the trends in Ni and Cr represent the interaction between domination of river discharge from the Južna

(lower Ni and Cr values) and Zapadna Morava (higher Ni and Cr), it may be used to trace the palaeo-river drainage system over the Central Balkan. Due to the different characteristics of its catchments, understanding of palaeo-river drainage system can be used to infer a signal of past precipitation change on a wider region (Supplementary Fig. 11). Under current conditions, the Zapadna and Južna Morava River catchments are affected by different precipitation regimes[1]. River discharge of the Zapadna Morava River is strongly influenced by the tributary Ibar River, which contributes with almost the same amount of water runoff as the Zapadna Morava. Their catchment areas receive annual precipitation above 800 mm, with some areas >1000 mm, especially in the higher mountains[1]. Thus, a decrease in the rainfall amount in the western sectors of the Balkan Peninsula will have a significant effect on those rivers discharge rate. Even a gentle decrease in precipitation has a considerable effect on the discharge since it will affect both the Ibar and Zapadna Morava rivers and their corresponding catchments. Contrary, the Južna Morava River catchment area is located in the rain shadow of high mountains related to the Zapadna Morava River catchment, and it is mainly characterized by annual rainfall amounts of 500-600 mm, with some parts of enhanced annual precipitation not exceeding 800 mm[1]. Since this catchment area is composed of only one major river as a main drainage course, decrease in already low precipitation will have much weaker influence. According to that, the discharge of the Južna Morava River would be less sensitive to precipitation changes over its catchment, while the discharge of the West Morava River would strongly depend on the precipitation regime over its catchment area. Therefore, higher values of Ni and Cr at the Stalać section would indicate more precipitation over the Zapadna Morava catchment relative to the Južna Morava catchment. Also, higher precipitation would enhance erosion processes over the Zapadna Morava catchment compared to the Južna Morava catchment, and therefore

additionally enhance the contribution of particles from its catchment area. Contrary, low values of Ni and Cr would indicate mostly similar distribution of the precipitation over these two catchment areas. Generally, it can be assumed that domination of Južna Morava River sediments (high Ni and Cr values) during glacials would indicate very low precipitation over both catchment areas. That would point to very dry conditions where the precipitations did not reach into the interior of Balkans. However, domination of particle from Južna Morava River over interglacials would indicate equally high precipitation over these two catchment areas. That means that air masses that bring precipitation crossed even over the high Dinaric Mountains west from the Južna Morava catchment.

7. Inferred past climate and environmental changes from the Central Balkan

Palaeosol S3 (MIS 9) is the strongest developed palaeosol at the Stalać profile for the studied time interval. High clay contributions, redness and vertic characteristics indicate strong weathering with hematite formation during dry and hot summers[43]. This indicates a strong influence of Mediterranean climate. Further to the south, referring to pollen-based palaeoenvironment reconstruction from Lake Ohrid[44], this period is characterized by the expansion of mesophilous vegetation, indicating warm and dry conditions for interglacial standards over the whole Balkans.

In general, the grain-size analysis is a well-accepted method in inferring palaeoclimate forcing upon the formation of loess-palaeosol sequences[45,46], where domination of coarse grain-size particles is usually associated with relatively strong winds and cold climate, while the domination of fine particles is associated with less strong wind, enhanced chemical weathering and warmer climate. Thus, the grain-size data from the following glacial (MIS 8, or loess L3) suggest a rather dry and cold environment

(Supplementary Fig. 5). High values of Ni and Cr (Supplementary Fig. 11) point towards dry conditions in south and central Serbia and possibly more moisture over mountainous areas to the west, as reflected by an enhanced particle contribution from the Zapadna Morava River catchment. Decrease in the percentage of coarse particles upwards points to warmer conditions, while gradual decrease in Ni and Cr values suggests slight increase in humidity over south and central Serbia during late MIS 8. Contrary to a benthic $\delta^{18}O$ isotope stack[24], our data suggest that the coldest and driest interval was the middle part of MIS 8 (rather than late MIS 8). However, this is in agreement with the MEDSTACK planktic ^{18}O data[47], as well as the Lake Ohrid pollen data[44].

The subsequent palaeosol S2 denotes another interglacial period that corresponds to MIS 7 and is much less developed compared to the S3. It still shows typical features of a palaeosol formed under warm and dry summer conditions, but it is less abundant in clay and with weaker redness values. Likely, Mediterranean climate influence still played an important role as determining factor for soil development during that time interval, but its influence is decreasing in comparison to MIS 9. Data from the Ohrid lacustrine record[44] shows a very high interglacial variability during MIS 7, with three expansions of trees interrupted by two intervals with herb expansion. It is more challenging to interpret palaeo-river drainage network and it's dynamics during interglacials. Higher contribution of the Južna Morava alluvium particles observed during all interglacials (Supplementary Fig. 11) likely indicates similar amounts of precipitation over Central Balkan, showing that althoug decerasing with time, Meditteranean influence was stronger than today during all three past interglacials. Alternatively, the closer Južna Morava valley might be favoured as a source area simply due to weaker wind dynamic

during interglacials. However, first scenario seems to be more plausible becouse of the vicinity of all river valleys suggested as souce areas.

The unit corresponding to the MIS 6 glacial period in the Stalać record is the L2. The abundance of sand in this unit implies strong wind dynamics and apparently the most severe conditions during past ~350,000 year. The geochemical tracing suggest that the L2 loess is mostly formed by particles from the Južna Morava River valley. This suggests very dry conditions over the whole Balkans. The Zapadna Morava River discharge was strongly reduced, whereas discharge from the Južna Morava catchment was not dramatically limited by the general aridization. According to grain-size distributions (Supplementary Fig. 5), the middle part of L2 was characterized by very dry and cold conditions, but at the onset and end of the glaciation environmental conditions may have been less severe. However, colour data (especially b* values; Supplementary Fig. 6) suggest different environmental conditions during the onset and the end of MIS 6. This is in agreement with the Lake Ohrid pollen data[44] which suggest a change from grassland (189–161 ka) to steppe dominated environment (161–126 ka).

The palaeosol representing the MIS 5 (S1) shows remarkably different features compared to the older palaeosols at the Stalać section. In the Middle Danube Basin palaeosols linked to the last two interglacials are represented by chernozem type soil with no remarkable difference between S2 and S1[17]. The Stalać section shows clear change in environmental conditions from a vertic layer in S2 to a chernozem-like S1. Similarities in genesis of S1 (MIS 5), L1SS1SSS2 and L1SS1SSS1 (MIS 3) pedocomplexes indicate broadly similar conditions during the last interglacial and MIS 3 interstadials at the Stalać section. High fine particle abundance (Supplementray Figs. 5 and 12) and high L* values (Supplementary Figs. 6 and 12) during the last glacial indicate relatively mild

conditions compared to the previous glaciation. The only proxy indicating enhanced weathering during the formation of S1 compared to MIS 3 is the frequency dependent magnetic susceptibility. However, frequency dependent magnetic susceptibility in general shows a narrow range of variations compared to other East European sections[39] and because of their different origin and properties compared to the Balkan loess, should be considered with caution. Conrary to the Stalać section, where MIS 3 palaeosol formations started well before 40 ka, palaeosols related to MIS 3 in the Middle Danube Basin are much weaker than interglacial S1 palaeosols, with the main soil formation phase after ~40 ka[5] and only very weak soil development before[2,4,5]. An exception is the L1SS1LLL1 layer, which shows the coarsest grain-size distribution (Supplementary Fig. 5), remarkably higher Cl values (Supplementary Fig. 7) and presence of volcanic glass shards (Supplementary Fig. 8). This clearly indicates a volcanic ash layer that we relate to the Campi Flegrei eruption at 39 ka that has produced widespread tephra deposits throughout the Balkans, lower Danube and northern Pontic area[20,21] (Supplementary Fig. 9). This layer most probably represents a short accumulation event of volcanic ash and aeolian silt, indicating strong adverse conditions during local impact of the Campanian Ignimbrite - Y5 tephra layer and possibly Heinrich event 4. However, more uniform and mild conditions during the late last glacial in Stalać are in agreement with results from another Central Balkans loess-palaeosol sequences section at Belotinac[48].

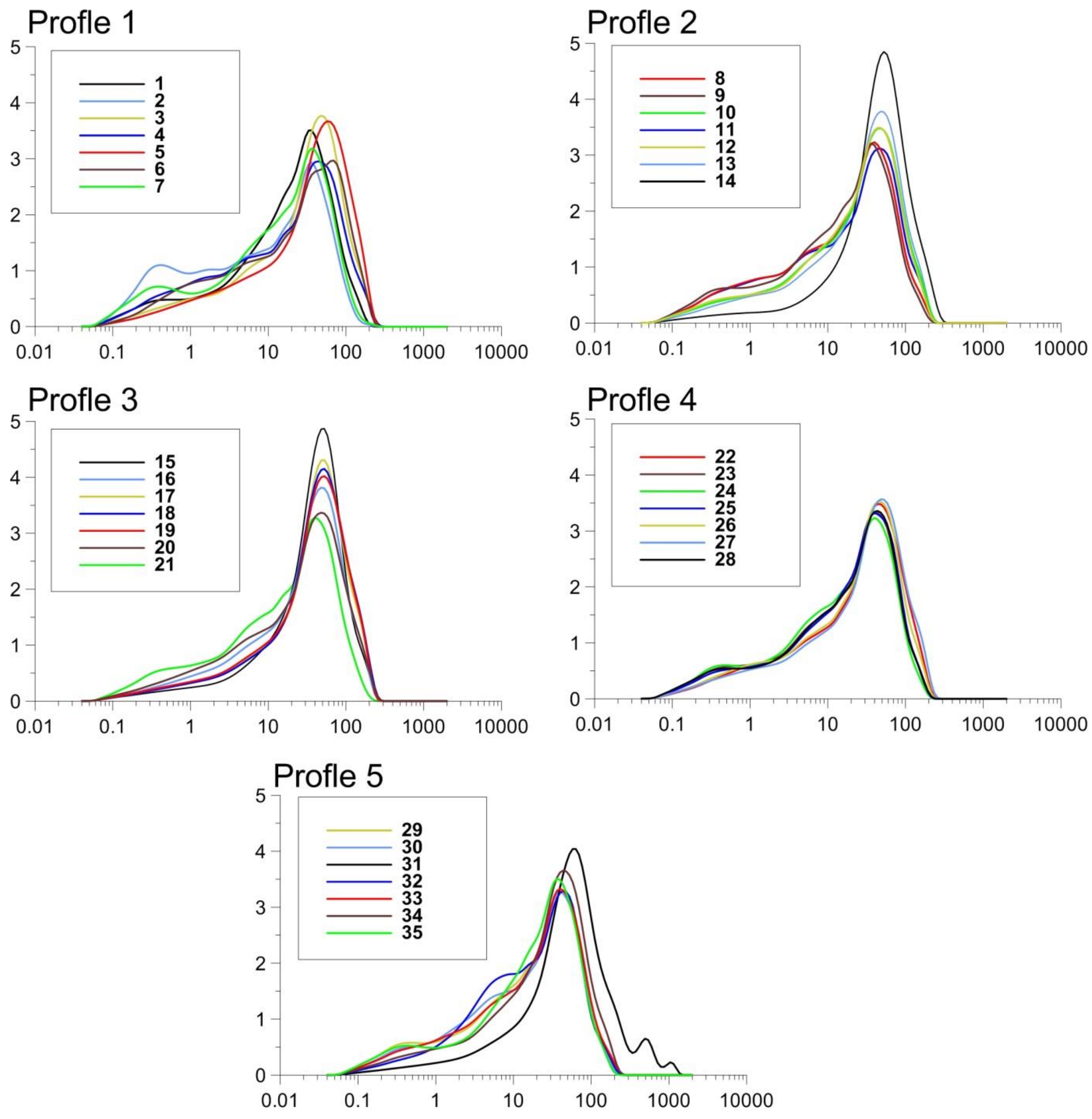

Supplementary Figure 10. Density distribution curves of grain size analyses of representative samples from all stratigraphic units at each sampled profile. Representative samples of one unit from each profile are presented by colour and codes that correspond to the numbers from 1 to 35. Explanation of each sample code is presented in Supplementary Table 6 where it is shown to which layer, MIS, and height on single and composite profile (if presented) the samples correspond.

Supplementary Table 6. Codes related to the samples presented in Supplementary Fig. 10. The first column contains the code, the second presents the layer and MIS to which the samples correspond, the third presents the height of the samples on original profile and the fourth column presents the height of the samples on the composite profile (if presented)

Code number	Layer / MIS	Height on profile [m]	Height on composite profile [m]
1	L4 / MIS 10	0.35-0.40	0.35-0.40
2	S3 / MIS 9	1.15-1.20	1.15-1.20
3	L3 / MIS 8	1.85-1.90	1.85-1.90
4	S2 / MIS 7	3.15-3.20	3.15-3.20
5	L2 / MIS 6	4.00-4.05	-
6	S1 - L1SS1 / MIS 5-3	4.50-4.55	-
7	S0 / MIS 1	5.40-5.45	-
8	S2 / MIS 7	0.20-0.25	-
9	S2 / MIS 7	0.70-0.75	-
10	S2LL1 / MIS 7	1.05-1.10	-
11	S2 / MIS 7	1.35-1.40	-
12	L2 / MIS 6	1.90-1.95	3.85-3.90
13	L2 / MIS 6	2.60-2.65	4.55-4.60
14	L2 tephra /MIS 6	2.90-2.95	4.85-4.90
15	L2 tephra / MIS 6	0.20-0.25	4.90-4.95
16	L2 / MIS 6	0.50-0.55	5.20-5.25
17	L2 / MIS 6	1.20-1.25	5.90-5.95
18	L2 / MIS 6	2.00-2.05	6.70-6.75
19	L2 / MIS 6	3.05-3.10	7.75-7.80
20	L2 / MIS 6	3.60-3.65	8-30-8.35
21	S1 / MIS 5	4.20-4.25	-
22	L2 / MIS 6	0.10-0.15	-
23	S1 / MIS 5	0.55-0.60	8.95-9.00
24	S1 / MIS 5	1.125-1.15	9.525-9.55
25	L1LL3 / MIS 4	1.375-1.40	9.775-9.80
26	L1LL3 / MIS 4	1.75-1.80	10.15-10.20
27	L1LL3 / MIS 4	2.05-2.10	10.45-10.50
28	L1SS2 / MIS 3	2.85-2.90	-
29	L1SS2 / MIS 3	0.35-0.40	11.35-11.40
30	L1SS2 / MIS 3	0.90-0.95	11.90-11-95
31	L1LL2 tephra / MIS 3	1.20-1.25	12.20-12.25
32	L1SS1 / MIS 3	1.85-1.90	12.85-12.90
33	L1SS1 / MIS 3	2.40-2.45	13.45-13.50
34	L1LL1 / MIS 2	3.10-3.15	14.10-14.15
35	S0 / MIS 1	3.85-3.90	14.85-14.90

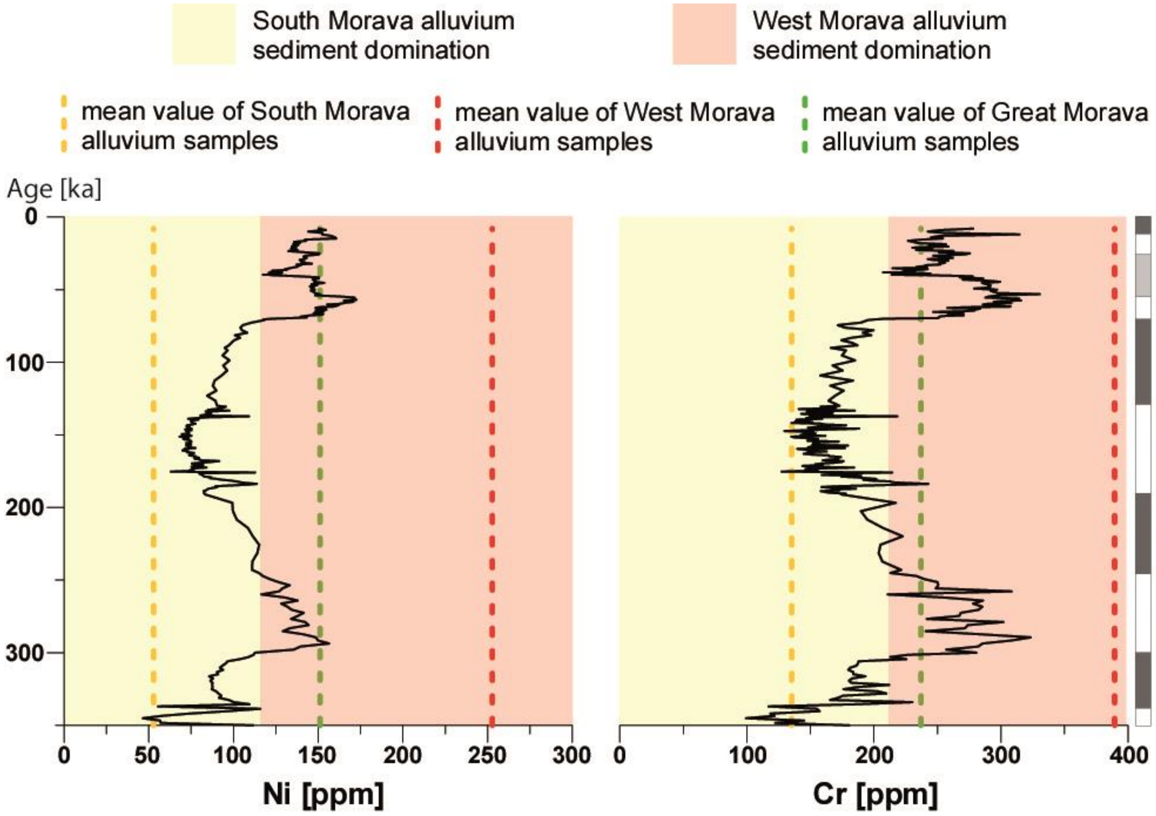

Supplementary Figure 11. Ni and Cr concentrations presented on age scale. Green dashed lines present mean value of samples from the Great Morava alluvium sediment, red dashed lines present mean value of samples from the Zapadna Morava alluvium sediment and yellow dashed lines present mean value of samples from the Južna Morava alluvium sediment. Values located in the light red rectangle are under relative domination of the Zapadna Morava alluvium as source area, while values in light yellow rectangle are under relative domination of the Južna Morava alluvium as source area.

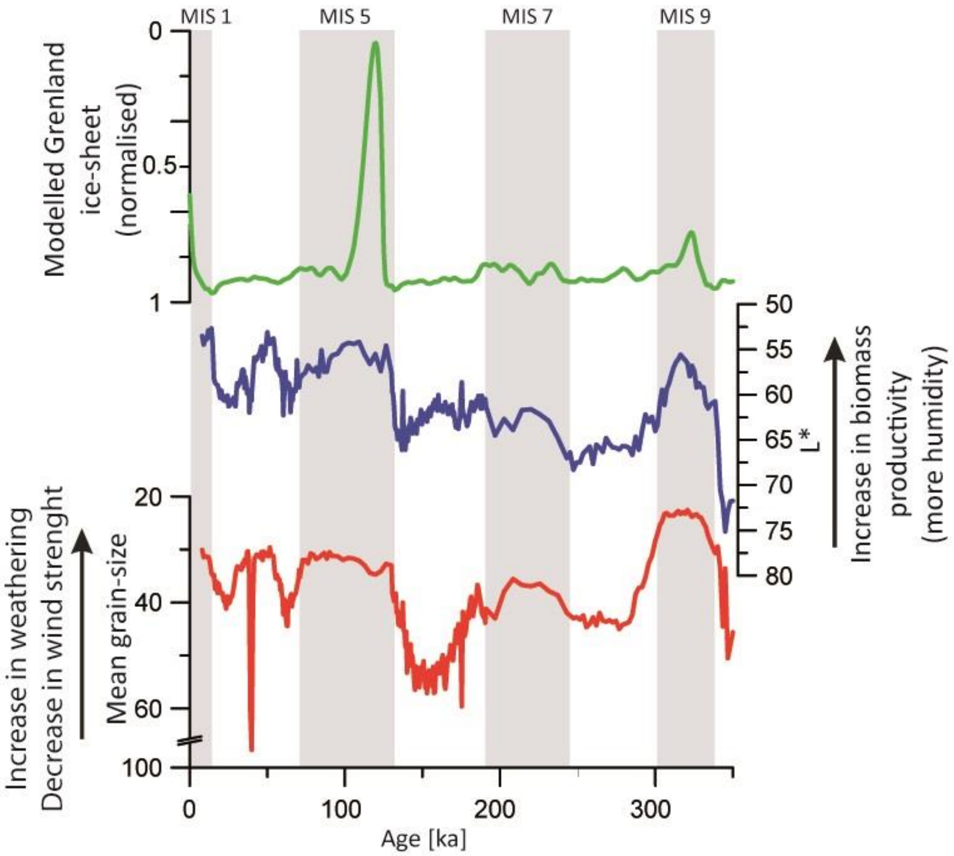

Supplementary Figure 12. Mean grain-size (μm) and L* values from the Stalać section compared to the normalized modelled Greenland ice-sheet volume[49]. Note that the transition from MIS 7 to MIS 6 at the Stalać section (transition from S2 to L2 layer) has to be considered with caution as explained in Supplementary chapter 4.

Supplementary references

1. Republic Hydrometeorological Service of Serbia. Available at: http://www.hidmet.gov.rs/eng/meteorologija/stanica_sr.php?moss_id=13383 (Accessed: 15th January 2016)
2. Fuchs, M. *et al.* Chronology of the Last Climatic Cycle (Upper Pleistocene) of the Surduk loess sequence, Vojvodina, Serbia. *Boreas* **37,** 66–73 (2008).
3. Hatté, C. *et al.* Excursions to C4 vegetation recorded in the Upper Pleistocene loess of Surduk (Northern Serbia): an organic isotope geochemistry study. *Clim Past* **9,** 1001–1014 (2013).
4. Schmidt, E. D., Machalett, B., Marković, S. B., Tsukamoto, S. & Frechen, M. Luminescence chronology of the upper part of the Stari Slankamen loess sequence (Vojvodina, Serbia). *Quat. Geochronol.* **5,** 137–142 (2010).
5. Stevens, T., Marković, S. B., Zech, M., Hambach, U. & Sümegi, P. Dust deposition and climate in the Carpathian Basin over an independently dated last glacial–interglacial cycle. *Quat. Sci. Rev.* **30,** 662–681 (2011).
6. Basarin, B. *et al.* Time-scale and astronomical forcing of Serbian loess–paleosol sequences. *Glob. Planet. Change* **122,** 89–106 (2014).
7. Buggle, B. *et al.* Stratigraphy, and spatial and temporal paleoclimatic trends in Southeastern/Eastern European loess–paleosol sequences. *Quat. Int.* **196,** 86–106 (2009).
8. Fitzsimmons, K. E., Marković, S. B. & Hambach, U. Pleistocene environmental dynamics recorded in the loess of the middle and lower Danube basin. *Quat. Sci. Rev.* **41,** 104–118 (2012).

9. Marković, S. B. *et al.* in *Climate Change: Relating the Astronomical Timescale to the Loess–Paleosol Sequences in Vojvodina, Northern Serbia* (eds. Berger, A., Mesinger, F. & Sijacki, D.) 65–78 (Springer Vienna, 2012).
10. Necula, C. & Panaiotu, C. Application of dynamic programming to the dating of a loess-paleosol sequence. *Romanian Rep. Phys.* **60,** 157–171 (2008).
11. Necula, C., Dimofte, D. & Panaiotu, C. Rock magnetism of a loess-palaeosol sequence from the western Black Sea shore (Romania). *Geophys. J. Int.* **202,** 1733–1748 (2015).
12. Kostić, N. & Protić, N. Pedology and mineralogy of loess profiles at Kapela-Batajnica and Stalać, Serbia. *CATENA* **41,** 217–227 (2000).
13. Marković, S. B. *et al.* Late Pleistocene loess-palaeosol sequences in the Vojvodina region, north Serbia. *J. Quat. Sci.* **23,** 73–84 (2008).
14. Marković, S. B. *et al.* Danube loess stratigraphy — Towards a pan-European loess stratigraphic model. *Earth-Sci. Rev.* **148,** 228–258 (2015).
15. Marković, S. B. *et al.* Environmental dynamics and luminescence chronology from the Orlovat loess–palaeosol sequence (Vojvodina, northern Serbia). *J. Quat. Sci.* **29,** 189–199 (2014).
16. Obreht, I. *et al.* Aeolian dynamics at the Orlovat loess–paleosol sequence, northern Serbia, based on detailed textural and geochemical evidence. *Aeolian Res.* **18,** 69–81 (2015).
17. Marković, S. b. *et al.* Loess in the Vojvodina region (Northern Serbia): an essential link between European and Asian Pleistocene environments. *Neth. J. Geosci.* **91,** 173–188 (2012).

18. Vandenberghe, J., Marković, S. B., Jovanović, M. & Hambach, U. Site-specific variability of loess and palaeosols (Ruma, Vojvodina, northern Serbia). *Quat. Int.* **334–335,** 86–93 (2014).
19. Constantin, D., Timar-Gabor, A., Veres, D., Begy, R. & Cosma, C. SAR-OSL dating of different grain-sized quartz from a sedimentary section in southern Romania interbedding the Campanian Ignimbrite/Y5 ash layer. *Quat. Geochronol.* **10,** 81–86 (2012).
20. Fitzsimmons, K. E., Hambach, U., Veres, D. & Iovita, R. The Campanian Ignimbrite Eruption: New Data on Volcanic Ash Dispersal and Its Potential Impact on Human Evolution. *PLoS ONE* **8,** e65839 (2013).
21. Veres, D. *et al.* The Campanian Ignimbrite/Y5 tephra layer – A regional stratigraphic marker for Isotope Stage 3 deposits in the Lower Danube region, Romania. *Quat. Int.* **293,** 22–33 (2013).
22. Morley, M. W. & Woodward, J. C. The Campanian Ignimbrite (Y5) tephra at Crvena Stijena Rockshelter, Montenegro. *Quat. Res.* **75,** 683–696 (2011).
23. Leicher, N. *et al.* First tephrostratigraphic results of the DEEP site record from Lake Ohrid, Macedonia. *Biogeosciences* **13,** 2151–2178 (2016).
24. Lisiecki, L. E. & Raymo, M. E. A Pliocene-Pleistocene stack of 57 globally distributed benthic δ18O records. *Paleoceanography* **20,** PA1003 (2005).
25. Bösken, J. *et al.* New luminescence-based geochronology framing the last two glacial cycles at the southern limit of European Pleistocene loess in Stalać (Serbia). Geochronometria, manuscript number: D-16-00045 (submitted).
26. Thiel, C. *et al.* Luminescence dating of the Stratzing loess profile (Austria) – Testing the potential of an elevated temperature post-IR IRSL protocol. *Quat. Int.* **234,** 23–31 (2011).

27. Galbraith, R. F., Roberts, R. G., Laslett, G. M., Yoshida, H. & Olley, J. M. Optical Dating of Single and Multiple Grains of Quartz from Jinmium Rock Shelter, Northern Australia: Part I, Experimental Design and Statistical Models*. *Archaeometry* **41,** 339–364 (1999).
28. Guerin, G., Mercier, N. & Adamiec, G. Dose-rate conversion factors: update. *Anc. TL* **29,** 5–8 (2011).
29. Brennan, B. J. Beta doses to spherical grains. *Radiat. Meas.* **37,** 299–303 (2003).
30. Mejdahl, V. Thermoluminescence Dating: Beta-Dose Attenuation in Quartz Grains. *Archaeometry* **21,** 61–72 (1979).
31. Huntley, D. J. and Baril, M.R. The K content of the K-feldspars being measured in optical dating or in thermoluminescence dating. Ancient TL 15, 11–12 (1997).
32. Anechitei-Deacu, V., Timar-Gabor, A., Fitzsimmons, K. E., Veres, D. & Hambach, U. Multi-method luminescence investigations on quartz grains of different sizes extracted from a loess section in Southeast Romania interbedding the Campanian Ignimbrite ash layer. *Geochronometria* **41,** 1–14 (2014).
33. Civetta, L. *et al.* Geochemical zoning, mingling, eruptive dynamics and depositional processes — the Campanian Ignimbrite, Campi Flegrei caldera, Italy. *J. Volcanol. Geotherm. Res.* **75,** 183–219 (1997).
34. Signorelli, S., Vaggelli, G., Francalanci, L. & Rosi, M. Origin of magmas feeding the Plinian phase of the Campanian Ignimbrite eruption, Phlegrean Fields (Italy): constraints based on matrix-glass and glass-inclusion compositions. *J. Volcanol. Geotherm. Res.* **91,** 199–220 (1999).
35. Pyle, D. M. *et al.* Wide dispersal and deposition of distal tephra during the Pleistocene 'Campanian Ignimbrite/Y5' eruption, Italy. *Quat. Sci. Rev.* **25,** 2713–2728 (2006).

36. Antoine, P. *et al.* High-resolution record of the last climatic cycle in the southern Carpathian Basin (Surduk, Vojvodina, Serbia). *Quat. Int.* **198,** 19–36 (2009).
37. Bokhorst, M. P. *et al.* Atmospheric circulation patterns in central and eastern Europe during the Weichselian Pleniglacial inferred from loess grain-size records. *Quat. Int.* **234,** 62–74 (2011).
38. Basarin, B. *et al.* The Belotinac section (Southern Serbia) at the southern limit of the European loess belt: Initial results. *Quat. Int.* **240,** 128–138 (2011).
39. Buggle, B. *et al.* Iron mineralogical proxies and Quaternary climate change in SE-European loess–paleosol sequences. *CATENA* **117,** 4–22 (2014).
40. Wang, X. *et al.* Deciphering magnetoclimatological patterns of late Early to early Middle Pleistocene loess–paleosol sequences in the western Chinese Loess Plateau. *Glob. Planet. Change* **130,** 37–46 (2015).
41. Evans, M. E., Rutter, N. W., Catto, N., Chlachula, J. & Nyvlt, D. Magnetoclimatology: Teleconnection between the Siberian loess record and North Atlantic Heinrich events. *Geology* **31,** 537–540 (2003).
42. Kabata-Pendias, A. *Trace Elements in Soils and Plants, Fourth Edition*. (CRC Press, 2010).
43. Torrent, J., Schwertmann, U., Fechter, H. & Alferez, F. Quantitative relationships between soil color and hematite content. *Soil Sci.* **136,** 354–358 (1984).
44. Sadori, L. *et al.* Pollen-based paleoenvironmental and paleoclimatic change at Lake Ohrid (south-eastern Europe) during the past 500 ka. *Biogeosciences* **13,** 1423–1437 (2016).
45. Hao, Q. *et al.* Delayed build-up of Arctic ice sheets during 400,000-year minima in insolation variability. *Nature* **490,** 393–396 (2012).

46. Vandenberghe, J. Grain size of fine-grained windblown sediment: A powerful proxy for process identification. *Earth-Sci. Rev.* **121,** 18–30 (2013).

47. Wang, P., Tian, J. & Lourens, L. J. Obscuring of long eccentricity cyclicity in Pleistocene oceanic carbon isotope records. *Earth Planet. Sci. Lett.* **290,** 319–330 (2010).

48. Obreht, I. *et al.* The Late Pleistocene Belotinac section (southern Serbia) at the southern limit of the European loess belt: Environmental and climate reconstruction using grain size and stable C and N isotopes. *Quat. Int.* **334–335,** 10–19 (2014).

49. de Boer, B., Lourens, L. J. & van de Wal, R. S. W. Persistent 400,000-year variability of Antarctic ice volume and the carbon cycle is revealed throughout the Plio-Pleistocene. *Nat. Commun.* **5,** 2999 (2014).
